# Airborne PCB Concentrations in Portland, Oregon: Emissions and Contributions from the Portland Harbor Superfund Site

**DOI:** 10.1021/acsestair.5c00244

**Published:** 2025-11-13

**Authors:** Alexis R. Slade, Andres Martinez, Martine E. Mathieu-Campbell, Cassie Cohen, Shannon Lea Watkins, Keri C. Hornbuckle

**Affiliations:** † Department of Civil and Environmental Engineering, IIHR-Hydroscience and Engineering, 4083 The University of Iowa, Iowa City, Iowa 52242, United States; ‡ Center for Geospatial Analytics, North Carolina State University, Raleigh, North Carolina 27695, United States; § Portland Harbor Community Coalition, Portland, Oregon 97211, United States; ∥ Department of Community and Behavior Health, College of Public Health, The University of Iowa, Iowa City, Iowa 52242, United States

**Keywords:** airborne PCB concentration, air−water emissions, flux, Portland Harbor, PUF−PAS, sampling rates, superfund site

## Abstract

We investigated airborne concentrations of polychlorinated biphenyls (PCBs) near the Portland Harbor Superfund Site (PHSS), a historical and culturally significant location. In collaboration with residents, we measured airborne PCBs using polyurethane foam passive air samplers (PUF–PAS) deployed for 6 weeks. Additionally, we estimated PCB emissions based on the flux calculations from Portland Harbor (PH) water using PCB concentrations reported by the U.S. EPA to predict airborne PCB concentrations with an atmospheric dispersion model (AERMOD). Measured airborne total PCB concentrations ranged from 70 to 910 pg m^–3^ with a geometric mean of 330 pg m^–3^, which is lower than concentrations observed in other known PCB-contaminated areas in the U.S. Air congener distributions resembled commercial Aroclor mixtures 1016 and 1242, and estimated PCB flux from the water averaged 450 ± 120 ng m^–2^ d^−1^. Predicted airborne PCB concentrations ranged from 1 to 124 pg m^–3^, with enrichment in non-Aroclor congeners when PH water is the sole source. However, all predicted concentrations were lower than measured values and exhibited different congener distributions, suggesting that PCB flux from PH water contributes only a minor portion (∼2%) of Portland’s airborne PCB burden, and that additional PCB sources exist within the community.

## Graphical abstract

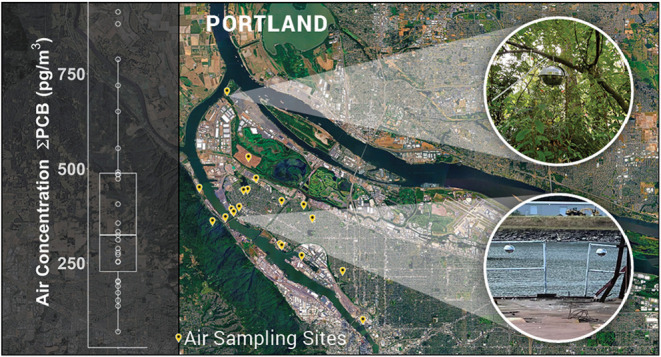

## Introduction

Polychlorinated biphenyls (PCBs) are anthropogenic chemicals that were industrially produced and widely sold as commercial mixtures until they were banned in the United States (U.S.) in 1979.^1^ Due to their widespread use and physical-chemical properties, PCBs can be transported in the atmosphere, water, and soil, depending on the temperature and their sorption to organic matter. As a result, they affect environmental systems, people, and wildlife both near and far from their source.^2–4^ The effects of PCB release from water bodies into the atmosphere have been widely studied in areas of concern such as the Great Lakes, and Superfund sites like the New Bedford Harbor, Hudson River, and internationally in India, and China.^5–9^ Like many other surface waters near industrial centers, Portland Harbor (PH) is contaminated with PCBs.^10,11^ A Superfund Site is a contaminated location designated under the Comprehensive Environmental Response, Compensation, and Liability Act (CERCLA) that poses risks to human health or the environment, where the United States Environmental Protection Agency (U.S. EPA) can oversee cleanup and require responsible parties to remediate or fund the remediation. Portland Harbor is listed on the U.S. EPA National Priorities List for cleanup and is referred to as the Portland Harbor Superfund Site (PHSS).^12^ It is surrounded by Portland, Oregon, a city of more than 2,200,000 people.

The communities surrounding PH, led by Portland Harbor Community Coalition (PHCC), were interested in the fate of PCBs at the PHSS and the potential for airborne dispersion into the City of Portland. Founded in 2012, PHCC consists of a collective of organizations and individuals representing the community who work collaboratively to advance their initiatives. The PHCC is the primary community-based organization through which community members express their concerns and contribute to decision making about the PHSS. PHCC is working to heal the polluted land, air, and water of the PH through elevating the voices of communities most impacted by pollution in the PHSS. Their mission is to ensure impacted communities benefit from and lead the cleanup, restoration, and redevelopment of the PH. Questions PHCC wanted to address included: What are the levels of PCBs in the surrounding community’s air? How far do PCBs travel from the PH to surrounding neighborhoods? Did U.S. EPA’s past modeling assessment represent contaminated conditions of the atmosphere surrounding the PH? Despite reports of PCBs in PH sediment and water, no studies have reported airborne PCB concentrations in the surrounding community.

In 2021, the U.S. EPA finalized agreements with responsible parties to implement the final cleanup plan for the PHSS encompassing river mile (RM) 1.9–11.8.^13^ Completion of remediation could take up to 13 years. Currently, this site is in the remedial design phase, which could take four years to complete. With continued remediation efforts to include planned dredging around the PH in 2027, the community was concerned that the site will become a source of increased airborne PCBs.^14,15^ As a team, PHCC and the Iowa Superfund Research Program (ISRP), an academic research center dedicated to the study of PCBs, hypothesized that PCBs are emitted from the harbor and that these fluxes are dispersed into the air of the City of Portland. We addressed this hypothesis using a three-pronged approach: first, by predicting PCB emissions from the harbor’s water; next, by assessing the extent of atmospheric dispersion; and last, by directly measuring airborne PCBs near PH. By comparing estimated and observed concentrations of airborne PCBs in Portland, we can evaluate the importance of the harbor as a PCB source. Here, we present both the scientific results of this collaboration and how the impact of Community-Engaged Research between the ISRP and PHCC provides scientific knowledge on the effectiveness of the cleanup process. This partnership also provides communities with meaningful participation to further ensure that PH becomes a minimal health risk.

## Methods and Materials

### Establishing Partnerships

The collective participation of the ISRP and PHCC was the result of a community centered approach to address PHCC’s question about ambient air concentrations of PCBs in their community.^16–18^ This project is unique in that the community enlisted the help of the ISRP to address their concerns.

PHCC worked with the ISRP to develop the central research question, identify sampling sites, manage air samplers, and involve stakeholders early to facilitate more resident involvement. We collectively collaborated over all phases of the project including planning, data collection, communicating project results to multiple parties through townhall meetings, presentations to nonprofit organizations and government agencies, and in reports.

### Site Identification

The PHSS – a 10-mile stretch of the Willamette River – is north of downtown Portland, and divides the city into east and west sides (Figure 1).^19^ The air sampling sites selected were within about a 5-km radius of the PH and are all classified as urban environments. Most of Portland’s terrain drains to the PH, also known as the Willamette River. The harbor is a heavily industrialized area that has housed the manufacturing of ships, petroleum, metal, and power-generating activities conducted by Arkema Inc., Evraz Inc., Schnitzer Steel Industries Inc., and the Marine Group, who were the four major contributors to the site’s contamination as identified by U.S. EPAs preremedial design investigation.^10,12^ Most of the residences surrounding the PH are single-dwelling homes.^20^ The PHSS also has historical and cultural significance. It was the site of shipbuilding and deconstruction before and after WWII, followed by various industries in subsequent years, some of which were part of the timber industry employing the largely working class population living on or near the site. There are still active industries surrounding communities, making it one of the most complicated Superfund Sites designated by the U.S. EPA.^21^ The harbor has been a source of aquatic recreation over the years including fishing and boating. It provides a critical migratory corridor and rearing habitat for Chinook salmon, and is home to endangered steelhead and green sturgeon.^22^ There is also a high population of people experiencing homelessness who live along the PH and utilize the harbor as a water source.^23^

**Figure 1:**
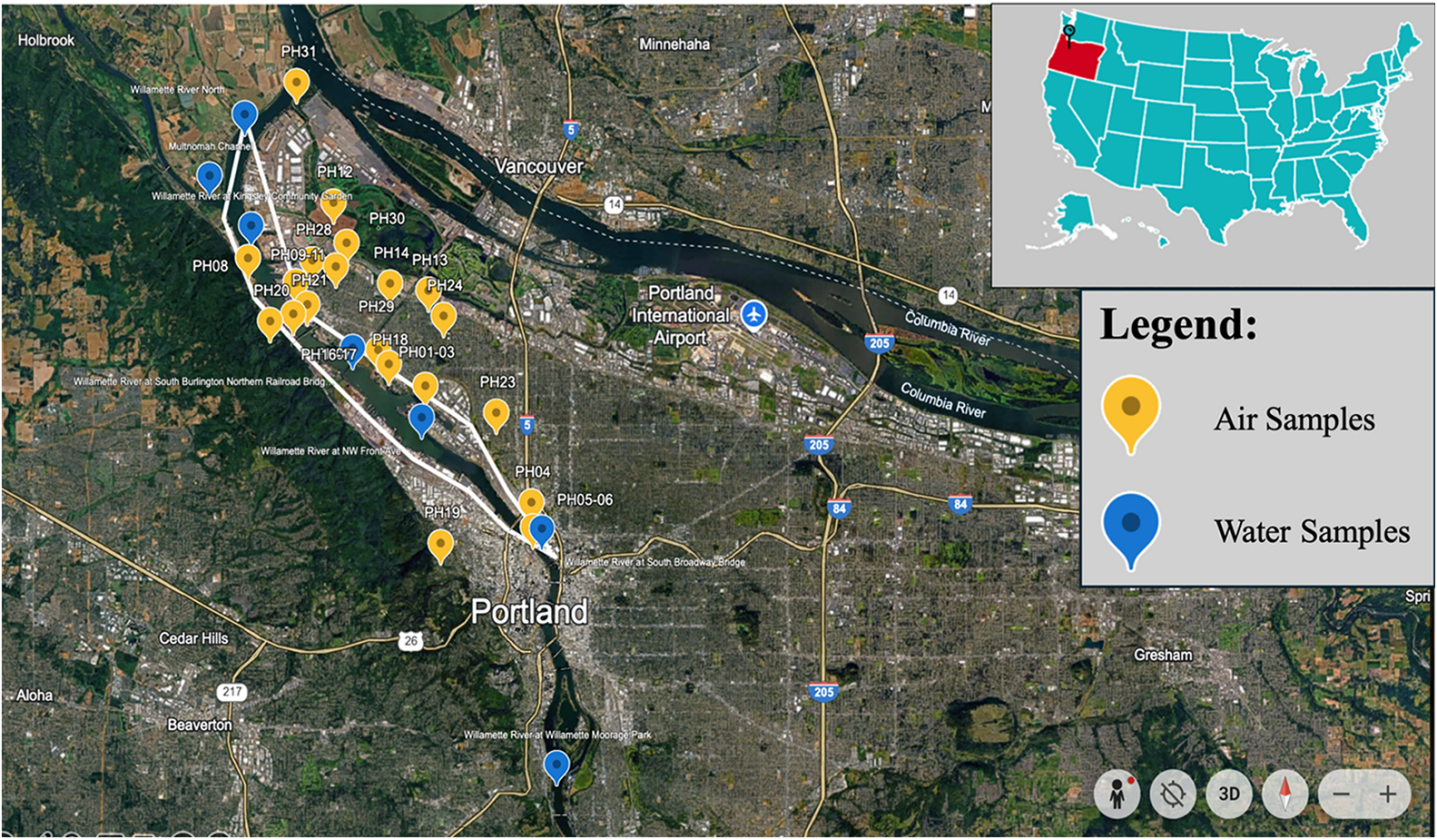
Map of the study area showing the locations of the air samplers (yellow points) and of the U.S. EPA-collected water samples (blue points) from 2018 to 2019. The white line outlines the overall PHSS boundary.

### PCB Flux Calculation and Emission Predictions from Portland Harbor

Congener-specific PCB flux from the water into the atmosphere surrounding the PH were calculated using PCB water concentrations obtained from U.S. EPA water quality monitoring reports from 2018 to 2019.^24^ This is the only and most recent PCB water concentration data for the PHSS. Analytical methods and quality assurance/quality control (QA/QC) for PCB water measurements are provided in the Supporting Information (SI). Twenty-one water samples were reported from 7 sites around the PH (Figure 1). All samples were collected from the lower Willamette River and in two upstream areas. Water sampling was conducted during high-flow, low-flow, and storm-flow events. We were interested in the worst-case scenario for the flux; thus, we averaged the three highest samples, located at the Willamette River North, NW Front Ave, and Kingsley Community Garden.

We used the entire superfund water area (15 km^2^), to calculate hourly gas emissions in g s^–1^ based on the flux calculations (ng m^–2^ d^–1^), for each congener using individual PCB congener air–water mass transfer coefficients and the concentration of dissolved PCBs in the PH water.^9,25,26^ Calculations have been described elsewhere.^6,27^ Various meteorological factors influence the rate and expanse of fluxes; therefore, data including air temperature, water temperature, and wind speed were used to calculate PCB flux from the PH. These data were obtained from the weather station at the Portland International Airport (Station WBAN:24229). The Portland International Airport, located approximately 10 km from the PH, was identified as the closest meteorological station representing the study area’s conditions, terrain, and land use. NOAA’s National Centers for Environmental Information (NCEI) Web site’s Integrated Surface Database (ISD) data sets, a global database that contains compiled surface observations, were used to gather wind speed information.^28^ Water temperature and atmospheric pressures were gathered from the NOAA National Buoy Center in the PH area (Station TLBO3).^29^ The overall PCB fluxes for each of the PCB congeners *F*_PCB_i__, ng m^–2^ day^–1^ was calculated using the gradient-flux law (eq 1):

1 $$F_{{\mathrm{PCB}}_{i}}=V_{{\mathrm{PCB}}_{i}a/w}\times C_{{\mathrm{PCB}}_{i}w}$$

where *V*_PCB_i_ a/w_ is the air–water mass transfer coefficient for the *i*th PCB (m d^–1^) and *C*_PCBi w_ is the concentration of the truly dissolved PCBs in the water column-those not bound to particles or organic matter- for the ith PCB (ng m^–3^). The air–water mass transfer coefficient was determined using a modification of the Whitman two-film model, which calculates individual velocities across the air and water films to derive the overall air–water mass transfer coefficient. The physical and chemical properties of individual PCB congeners used in the mass transfer coefficient calculations were adjusted for environmental and meteorological conditions. Once the flux was determined, it was multiplied by the water surface area to estimate emissions, which were then used as inputs for AERMOD. Detailed calculations are provided in the SI.

### Atmospheric Dispersion Modeling of PCB Concentrations Using AERMOD

We used our calculated flux multiplied by the area of the study location, meteorological inputs such as wind and temperature, and flat terrain characteristics to estimate airborne PCB concentrations and emissions at downwind receptor locations.^30,31^ The primary purpose for using water-derived fluxes in AERMOD was to examine whether these fluxes could explain the airborne PCB concentrations we measured around the PH and surrounding neighborhoods. This study used the steady-state Gaussian plume model, AERMOD, to analyze an area encompassing a 17 km radius around the Willamette River (Center reference point: longitude: 122° 39′ 48.50″W, latitude: 45° 34′15.67″N). This same approach has been used, particularly for area sources, to predict PCB concentrations in Lake Michigan; East Chicago, Indiana; and in New Bedford Harbor Superfund sites.^5,6,32,33^ Since the flux was calculated using data from August 2018, the meteorological conditions during that period were assessed and compared to those of the air sampling period in 2022. A 26.5 km stretch of the river, extending from Northwest to Southwest, where the water samples were collected, was identified as the PCB source area for this model (Figure 1). We used hourly meteorological surface data from the ISD, and upper air data from the Radiosonde Database that collects atmospheric measurements from radiosonde instruments.^34^ The Portland International Airport was used to obtain the ISD data, while the upper air data were obtained from the Salem McNary field station, located about 75 km from the study area. Meteorological data for wind parameters were sourced using the Automated Surface Observation Systems (ASOS) 1 min and 5 min data.^34^ These ASOS data were retrieved from the Local Climatological Data product. All meteorological data were processed using AERMET (version 12.0.0) and corresponded to the field sampling period from July eighth to August 17th, 2022.

The 30-m resolution land cover data from the 2016 National Land Cover Database (NLCD) were processed with AERSURFACE to determine land surface characteristics values of albedo, Bowen ratio, and surface roughness length.^35^ We defined a uniform 1.6 km resolution Cartesian grid receptor, for which elevation information was estimated. Then, we used the 21 airborne PCB field measurement sites as “sensitive receptors” to obtain PCB concentrations at these specific locations. In AERMAP, terrain elevations at each receptor point were estimated after preprocessing the 10-m resolution National Elevation Database.^35^ All the processed input data were incorporated into AERMOD View (v.12.0.0) to obtain spatial predictions of PCB concentrations for the 6-week study period.

### Air Sampling Measurements

We used polyurethane passive air samplers (PUF–PAS) to collect airborne PCBs.^6,36,37^ Before the deployment of the PUF–PAS, the PUF was cleaned using a 1:1 mixture of hexane:acetone (Dionex ASE 350).^38^ Each clean PUF disk was spiked with 25 ng of depuration compounds (DCs) ^13^C-PCB 28, 111, and 178.^5,39,40^ Because these compounds are not found in the environment, they were used as surrogates for determining the site-specific sampling rate of the PUF–PAS.^39,40^ See details in the SI.

Airborne PCB samplers placed in residential areas were secured on trees, backyards, patios with open airflow, and piers. At other sites, samplers were secured on ship decks, fences, near train tracks, in open fields and parks, and directly over water (Figure S1). There were five sites where samples were placed in triplicate and five field blanks were used at random sites around the harbor. Field blanks were exposed to the air then immediately sealed in aluminum foil and a plastic bag but otherwise exposed to all the steps in the sampling and analysis process, including transport and deployment.

After 6 weeks, we collected samples over two days. To prevent contamination, we collected and individually wrapped the PUFs in aluminum foil and Ziploc bags. We transported them, secured with ice packs, back to the University of Iowa’s lab. One of the sampling sites had three PUF–PAS stolen and one sampler from another site was disturbed rendering it unusable, so we obtained 28 of 32 samples. We stored the samples in a - 23 °C freezer until extraction and analysis were performed.

### Calculation of Atmospheric PCB Concentration

We used the PUF–PAS Sampling Rate Model interface pufpasvolume.org to obtain sampling rates (R_s_, m^3^ d^–1^) and effective volumes (*V*_eff_, m^3^).^41^ This model requires site-specific values such as sampler height, latitude and longitude, and deployment dates and accounts for different wind speeds and air temperatures using data from the Modern Era Retrospective-Analysis for Research and Applications (MERRA) and any selected deployment time. The model considers the physical-chemical properties of all 209 PCB congeners for calculations, the wind speed, atmospheric partial pressure, and temperature at 2 m above ground.^36,42^ The local wind speed and temperature for our samplers were not fully captured by the PUF–PAS modeled meteorology because our samplers were often placed in a protected location, including under eaves and trees. The micrometeorology experienced by each sampler affects their *R*_s_ and *V*_eff_. To account for this uncertainty, we used DCs according to the method of Moeckel et al.,^39^ to estimate sampler-specific local *R*_s_ and *V*_eff_. Details of DC calculations are presented in the SI. The utility of the DCs for determining *R*_s_ required that 20 to 80% of the spiked DC compounds be lost to volatilization during sample deployment. Only one of our DC compounds, ^13^C-PCB 28, met these criteria, and only for 13 of the 28 samples (Table S2).^36,40,42^ No loss of DC was found in 14 samples, rendering the DC method unreliable for estimating *R*_s_. We did find the information from the recovery of DC compounds useful for estimating the uncertainty in our determination of *R*_s_: we used the DC results from the 13 samples to account for the variability in the PUF-Sampling Rate Model. The PUF–PAS Sampling Rate Model interface predicted an *R*_s_ value of 4.9 m^3^ d^–1^ for PCB 28 for all sampling sites whereas the DC method R_s_ averaged 3.5 m^3^ d^–1^ for the same congener and ranged from 1.4–7.6 m^3^ d^–1^. This variability in the sampling rates resulted in a coefficient of variation (COV) of 72% across the 13 sites.

### Extraction and Analysis

We prepared the extraction of PUF samples containing PCBs and DCs by first spiking them with 10 ng of each surrogate standard (^13^C-labeled PCBs 3, 15, 31, 52, 118, 153, 180, 194, 206, and 209), then utilized a Dionex ASE-350 with a 1:1 acetone-hexane solution mixture under high pressure and temperature for extraction.^39,43,44^ The extracts were cleaned through a glass column containing sulfuric acid silica gel to obtain eluate which was then concentrated to ∼0.5 mL, which was then spiked with 10 ng of internal standard PCB30-d5 (2,4,6- trichlorobiphenyl-2′,3′,4′,5′,6′-d5, deuterated) and PCB204 (2,2′,3,4,4′,5,6,6′-octachlorobiphenyl). Tandem Mass Spectrometry GC-MS/MS (Agilent 7000) in multiple reaction monitoring mode was used to analyze the DCs and quantify 209 individual or coeluting congeners, in 173 chromatographic peaks (see Table S3).^37,40,45^ The GC was equipped with a Supelco SBP-Octyl capillary column (Poly­(50% *n*-octyl/50% methyl siloxane), 30 m × 0.25 mm ID, 0.25 μm film thickness) with UHP helium as the carrier gas (0.8 mL min^–1^) and UHP nitrogen as the collision gas (1.5 mL min^–1^).

### Quality Assurance, Control and Statistical Analysis

The data quality was evaluated using lab and field blanks, duplicates, and surrogate standard recoveries.^32^ In addition, NIST, SRM 2585 House Dust was used to evaluate method accuracy and precision. We extracted and analyzed five SRM samples using the same method used for air samples. The percent recovery of our measured values compared to certified values for the 28 PCB congeners reported yielded a mean of 89 ± 37% (Table S4). Surrogate standard recoveries were analyzed to account for sample losses during extraction and cleanup to show precision and correct for extraction efficiency. The mean and standard deviation of the surrogate standard recoveries was 88 ± 13% (Table S5) and sample PCB masses were corrected for recoveries below 100%. Triplicate samples of two separate sites in the field were used to show reproducibility and sample representativeness. The COV for triplicate samples (*n* = 2) was 46%. Sample representativeness was assessed by using a total of seven lab blanks and five field blanks. At least one of each was used per batch extraction. The blank masses were used to calculate the limit of quantification (LOQ) as the upper limit of the 99% confidence interval for the log_10_-transformed mass in the blanks, accounting for variability within the blank samples and any nonanalyte signals. The congener-specific LOQ ranged from 0.04 to 25 pg/PUF (Table S6).

All statistical analyses were performed in R (Version 2023.03.1 + 446). PCB flux calculations were performed incorporating a Monte Carlo simulation to estimate uncertainty in the determination of congener-specific fluxes from water. To compare the data of two related groups that did not follow normal or log-normal distribution, we employed a Wilcoxon nonparametric test for samples in different vicinities of the sampling. For spatial distribution analysis, we used two approaches: (i) regression of PCB concentrations against distance to the water, using a linear model with log_10_-transformed distance as the sole covariate; and (ii) Kriging interpolation to map spatial distribution and evaluate spatial correlation. The model’s accuracy and spatial autocorrelation were evaluated using Moran’s I, along with *R* ^2^ and root-mean-square error (RMSE) metrics. For the latter, we used the gstat package in R, and mapping was carried out in QGIS (version 5.15.13). Similarity analysis (cosine theta, cos θ) for PCB congener signals measured in air was also conducted using R, package lsa. Cos θ is the cosine of the angle between two multivariable vectors, which in the case of this study would be PCB congener profiles; a value of 0.0 indicates that the two profiles are completely different and 1.0 describes two identical profiles.^46^

## Results and Discussion

### Airborne PCB Concentrations

∑PCB atmospheric concentration in the PH ranged from 70 to 910 pg m^–3^, followed a log-normal distribution with a geometric mean of 330 pg m^–3^, and a median of 320 pg m^–3^. Most samples fell in the range of 70 to 750 pg m^–3^, where only three samples were above 750 pg m^–3^ (Figure S3). Airborne PCB concentrations measured at the sampling sites are higher than ambient air PCB concentrations measured in Seattle Washington, another urban location in this region of the U.S. Comparing results of this study to other studies is challenging because different sampling methods are used and different PCB congeners are reported. The Lower Duwamish Waterway Superfund study in Washington State utilized similar sampling and analytical methods to this study, but only analyzed and reported the concentrations of 27 PCB congeners which ranged from 180 to 280 pg m^–3^ (*n* = 4).^47^ Further, our measured PCB concentrations in PHSS are lower than those reported at more highly contaminated Superfund sites outside the region, such as New Bedford Harbor, Massachusetts. For example, our previous study, which used the same sampling and analytical methods for all 209 PCB congeners, reported concentrations ranging from 400 to 38,000 pg m^–3^ (*n* = 54).^6^

We examined the potential impact of the harbor on our findings by comparing samples collected near and far from the water, and by developing a linear regression model using distance to the water as the only covariate. There are statistically significant differences (*p* < 0.005) between the PCB concentrations of sampling sites placed directly above the water, with a median concentration of 788 pg m^–3^ (sites: PH9-12 and 21), compared to sites more than 2200 m away from the PH, with a median concentration of 196 pg m^–3^ (sites: PH13-14, 19 and 30). Sampling sites directly over the PH water and in community neighborhoods within 200 m of the harbor were enriched in PCBs 4, 8, 11, 18 + 30 and 52 relative to other samples, with PCB 8 showing a geometric mean of 19 pg m^–3^ and PCB 52 being 14 pg m^–3^. The linear model, with log_10_-transformed distance to the water as the predictor, explained 45% of the overall ΣPCB variability (Figure 2, top left). PCBs 45 + 51 exhibit the same trend with a 38% correlation (Figure 2, bottom left). Conversely, PCB 11 and 68, classified as non-Aroclors, showed a constant trend, indicating that distance is not a significant factor in their concentrations (Figure 2, right top and bottom).

**Figure 2:**
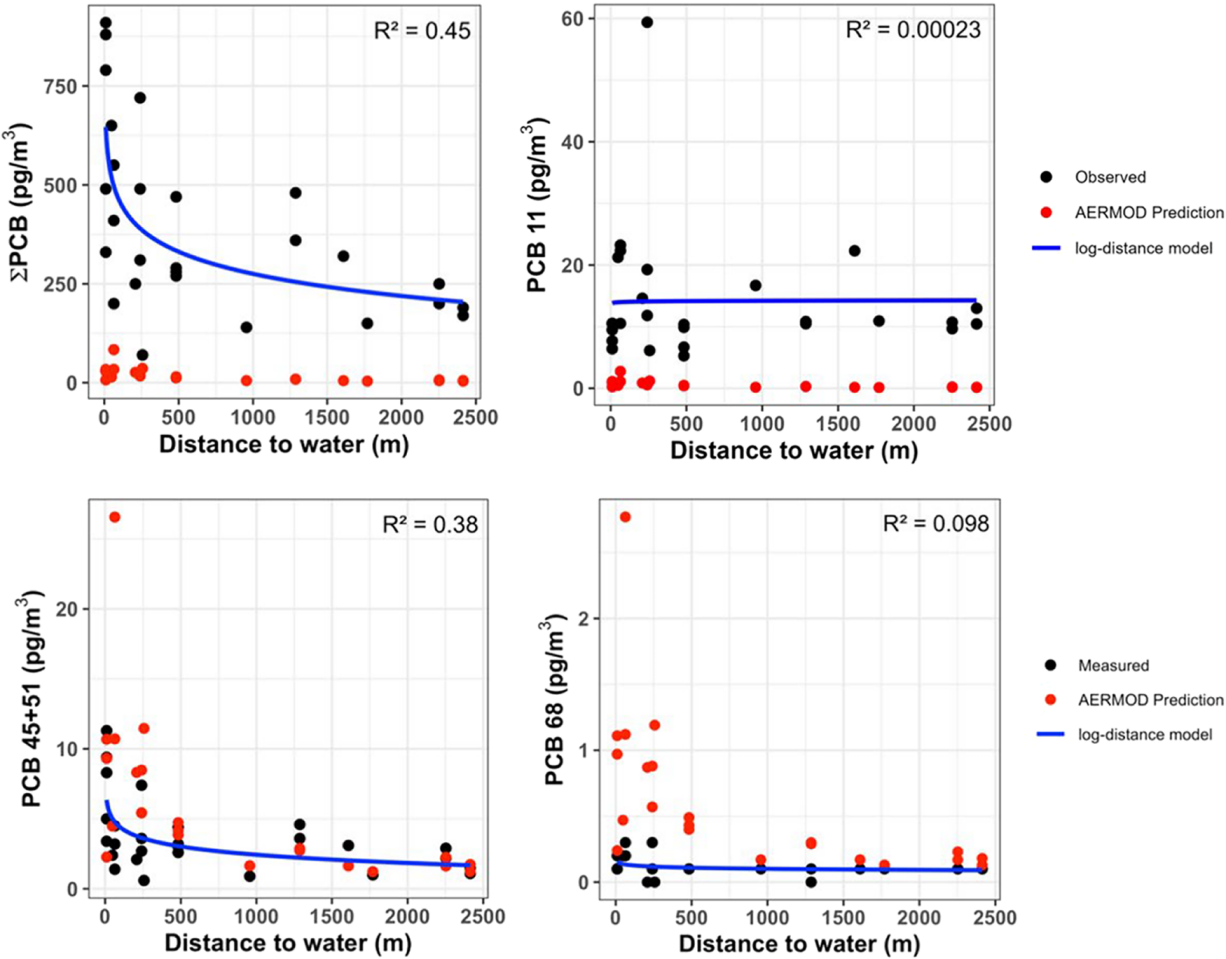
Airborne concentrations of total and selected PCBs versus distance from the water to urban communities. Black dots represent measurements from PUF–PAS, red dots are AERMOD predictions, and the blue line indicates the fitted linear regression model.

Two sampling sites (PH06 and PH31) did not follow the trend of higher concentrations, even though they were located near PH (∼260 m). We suspect that these low values are related to micrometeorological conditions affecting the two sites, which directly influence concentration calculations.^37,39^ The samplers at these locations were placed in trees (PH31) or next to houses (PH06), where airflow was minimal or absent. Because we used the PUF–PAS Sampling Rate Model interface, which does not fully capture site-specific wind conditions, the model likely overestimated the sampling rates, resulting in underestimation of PCB concentrations.^37,48^ However, we were able to calculate sampling rates for these two sites using DCs, which produced much lower values than the model estimates: 1.07 and 0.05 m^3^ day^–1^.^39^ These rates are more typical of indoor environments.^49^ The second sampling rate (0.05 m^3^ day^–1^) could not be reported because over 95% of the DCs remained in the PUF after sampling, falling outside the acceptable 20–80% lost range required for valid estimates.^37^ These findings helped us better interpret the PUF–PAS results and provided insight into the lower concentrations observed at these two sites.

Kriging predictions were consistent with our linear model, showing spatial correlation for ∑PCB, PCBs 45 + 51, and Aroclor congeners, such as PCBs 4, 8, 18 + 30, and 31, but no significant spatial structure for PCBs 11 and 68. Specifically, ∑PCB concentrations displayed significant spatial autocorrelation (Moran’s I = 0.095, *p* = 0.007) with moderate predictive performance when model predictions were tested against observed values not used in model fitting (*R* ^2^ = 0.28, RMSE = 160 ng m^–3^). PCBs 45 + 51 and 18 + 30 had stronger spatial structure (Moran’s I = 0.15 and 0.22, *p* < 0.001) with better predictive skill (*R* ^2^ = 0.41, RMSE = 1.01 ng m^–3^; *R* ^2^ = 0.48, RMSE = 10.6 pg m^–3^, respectively), while PCBs 4, 8, and 31 exhibited high spatial autocorrelation (Moran’s I = 0.23–0.24, *p* < 0.00001) and good predictive performance (*R* ^2^ = 0.44–0.53, RMSE = 7.2–11 pg m^–3^), indicating that Kriging effectively captured spatial patterns for total and selected individual PCB congeners with moderate-to-good accuracy. Consistent with our earlier analysis, the highest PCB concentrations were observed near the harbor, whereas the northern and eastern regions of the study area exhibited comparatively low levels. The predicted concentrations maps are shown in Figure 3.

**Figure 3:**
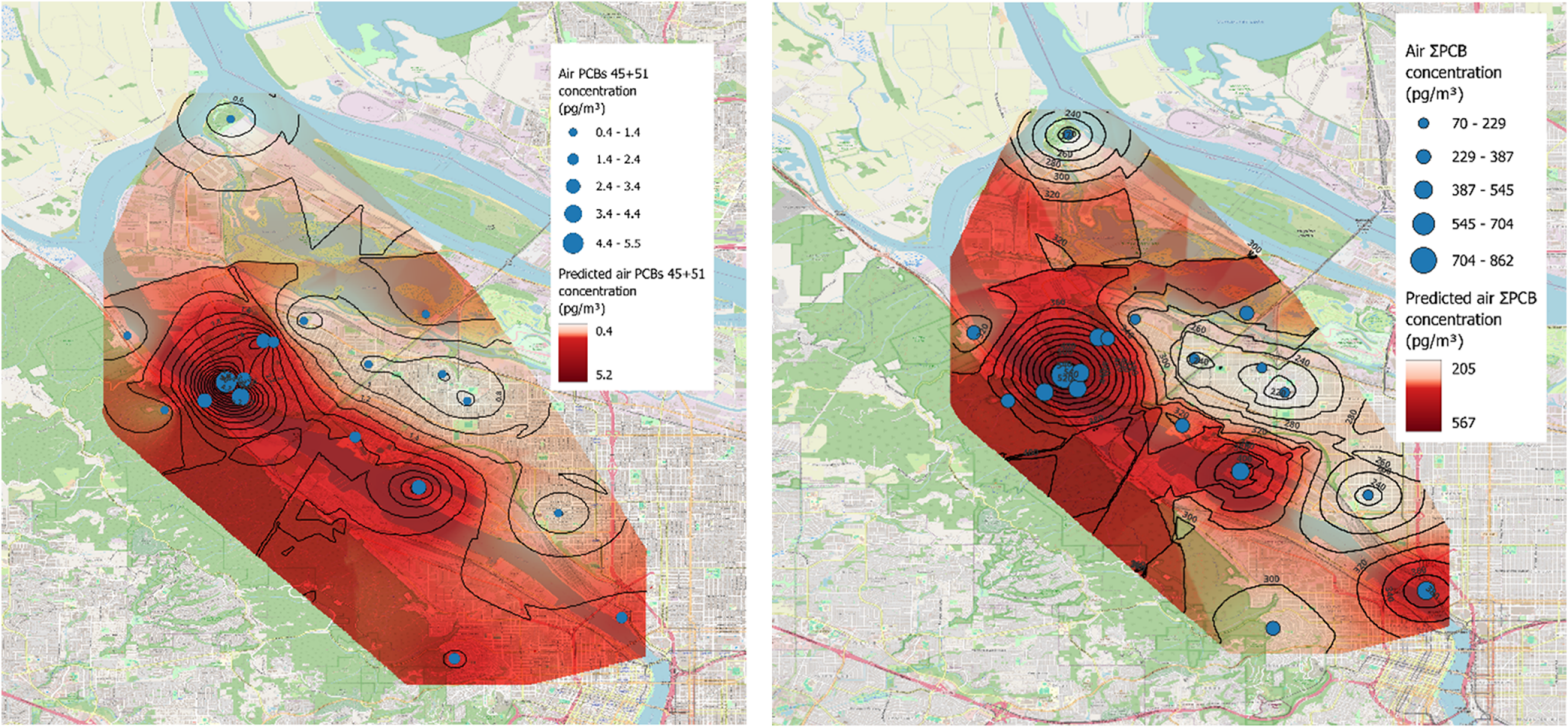
Kriging-predicted spatial distribution for PCBs across the study area: PCBs 45 + 51 concentration (left) and ∑PCB concentrations (right).

### PCB Congener Distribution in Air

Using cos similarity measures, PCB air sample profiles were compared between sampling sites and were found to be similar to each other with a cos θ = 0.84 ± 0.12. PCB site profiles were compared to reported Aroclor profiles, and most were found to be similar to Aroclor 1016 (cos θ = 0.74 ± 0.15) and Aroclor 1242 (cos θ = 0.78 ± 0.14). This aligns with the historical presence of carbonless paper mills at the PH, which used PCBs as ink pigments in carbonless copy paper, as well as other industries such as gas storage facilities, power plants, electronics manufacturers, and shipyards, each known to have used products containing Aroclor 1242.

The average PCB air profile when compared to the average water sample profiles show little similarity (cos θ= 0.34) (Figure S4). The congener profiles we measured in the air (Figure 4A) of the PH do not resemble the profiles of the PH water (Figure 4B), and the calculated flux (Figure 4C). This may be indicative of several possibilities. First, the profile of PCBs in the water is distinctly non-Aroclor, yet the surrounding sediment is enriched in Aroclors as reported by Megson et al.^50^ Second, PCBs from other local sources dominate the currently observed air concentrations around the PH.^14^ While the air samples contain mostly PCBs 4, 8, 11, 18 + 30, and 52 (Figure 4A), the water profiles have more PCBs 44 + 47 + 65, 45 + 51, and 68, which are byproducts of silicone rubber manufacturing (Figure 4B).^51^ For example, congeners with non-Aroclor sources such as paints/pigments (PCB 11), and products of biological metabolism (PCB 4) were important contributors to the total PCB concentrations in many air samples and contribute up to 53% of the PCBs whereas silicone (PCB 44 + 47 + 65, 45 + 51, and 68) was the dominant PCB congeners in the water samples with 48% contributions.^52^

**Figure 4:**
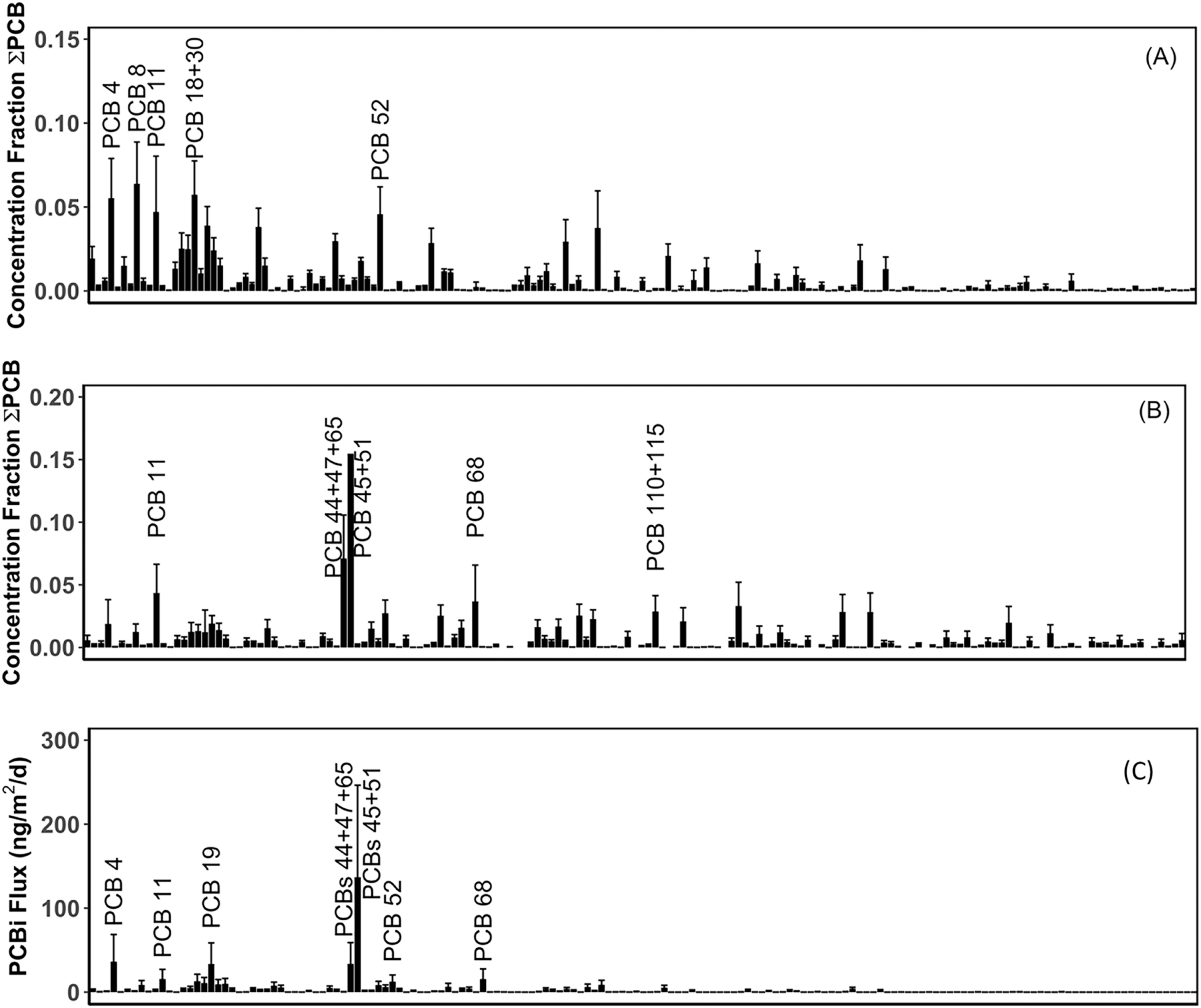
PCB congener profiles: (A) Average airborne concentrations fraction for the study area around the PH; (B) U.S. EPA-obtained water concentrations from 2018 to 2019. Locations of water samples were near PUF–PAS sampling sites (Figure 1); (C) Congener-specific fluxes from the PH. ΣPCB fluxes were calculated from the PH in ng m^2^ day^–1^, using U.S. EPA water data and meteorological data from the Portland International Airport.

### PCB Flux

The U.S. EPA water samples collected in August 2018 contained concentrations that ranged from 80 to 184 pg L^–1^, with an average of 132 ± 42 pg L^–1^. Between the time of water sampling and our air measurements, no major navigational projects occurred on the river that would likely alter concentrations. Our estimated flux of ΣPCBs averaged 450 ± 120 ng m^–2^ d^–1^. PH fluxes are higher compared to the Lower Duwamish Waterway study of 68 ng m^–2^ d^–1^, but lower than those of other known PCB-contaminated waters due to the comparably low dissolved-phase PCB congener concentrations reported for PH water.^6^ These flux calculations were used as input to predict the atmospheric dispersion of PCBs in AERMOD. We assume a constant and continuous average flux of 450 ng m^–2^ d^–1^.

### Atmospheric Modeling of PCB Dispersion and Concentrations

Figure 5 shows the interpolated spatial dispersion of PCB concentrations for the 6-week sampling time in 2022. The predicted concentrations varied from 1 to 124 pg m^–3^ with the dispersion following the wind direction profile (Figure S6a). Hence, for our study we found that people closer to the harbor were generally being exposed to higher PCB concentrations, which aligns with the relatively flat topography (less than 500 m), specifically near the source. This result is also similar to what is seen in the linear model and Kriging prediction. Evaluating characteristics such as calculated flux, 2022 meteorological inputs, and terrain characteristics strengthens our confidence in using 2018 flux calculations as a reasonable surrogate for 2022. A comparison of wind speed and direction revealed that PH experienced almost similar wind speed and direction for the same period in 2018 and 2022 (Figure S6a). The wind speed ranged from 3.60 to 8.80 m sec^–1^ and was observed approximately 30% of the time in 2018 and 33% of the time in 2022. Similarly, the time series analysis of temperature for 2018 and 2022 displayed the same pattern, except for a few days (specifically 07/13-07/21), with a daily average of 23 °C (Figure S6b). Figure S6c shows that daily average PBL height was greater than 800 m most of the days for the same time period in 2018 and 2022, with a third quartile of 911.80 and 844.00 m in 2018 and 2022 respectively. However, a lower PBL height were observed for a few days in 2018 compared with 2022, specifically during the ninth to the 11th and the 13th to the 17th in August (Figure S6c **)**. Additionally, as expected, the time series analysis revealed that for both years, PBL height was much lower in the morning (143.29 and 154.10 m at 7 am on average for 2018 and 2022 respectively) compared with the evening (1166.35 and 1163.82 m at 7 pm on average for 2018 and 2022 respectively).

**Figure 5:**
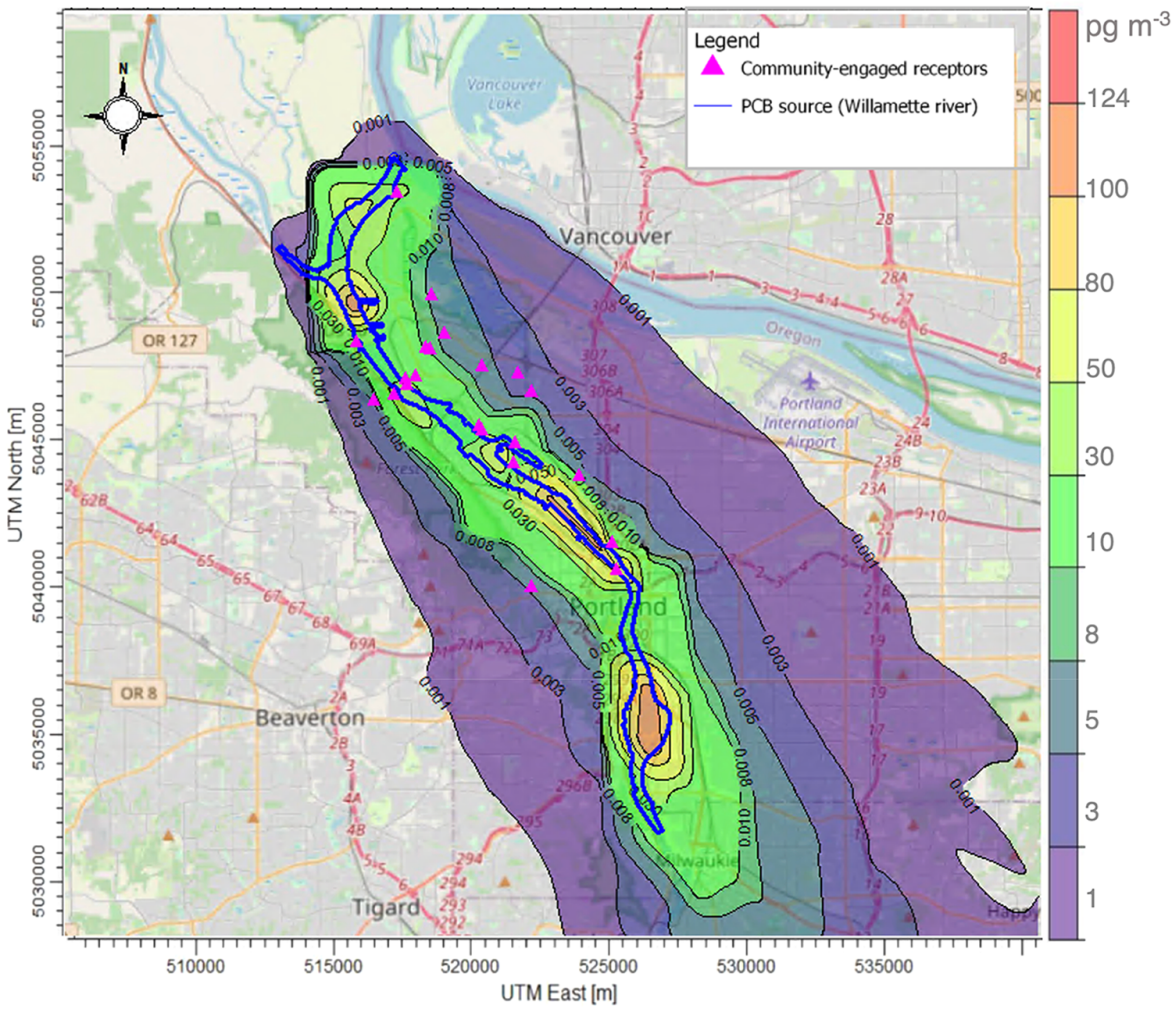
AERMOD dispersion map of the highest mean ΣPCB concentrations (ng m^–3^) from July to August 2022. Triangles represent community-engaged receptors (our PUF–PAS samplers) and the blue line is the PCB source (Willamette River).

We compared predicted concentrations from AERMOD with the measured concentrations from passive samplers at all sampling sites. The AERMOD-predicted airborne PCB concentrations were calculated for the entire sampling period, allowing a direct comparison with our measurements. The predicted concentrations of PCBs at the PH sampling sites ranged from 5 to 100 pg m^–3^ with median of 20 pg m^–3^. The average AERMOD predicted concentrations were 10% of the measured values at all sites (Table S7), suggesting a low contribution of the PH water emissions to our air measurements. When simulating the uncertainty of using a constant flux on airborne concentrations using the average plus and minus standard deviation of flux, sites PH06 and PH31 (Figure S3) show that the measured concentrations match the AERMOD model. However, as shown in Figure 2 (top left), the linear regression model produces a trend comparable to the AERMOD-predicted ∑PCB values, but with predicted concentrations approximately an order of magnitude lower. This similarity in trend suggests that AERMOD is capturing the atmospheric behavior and spatial distribution of PCBs, but the emissions used in the AERMOD may be underestimated. A final explanation is the presence of other sources, potentially unknown, that volatilized in a similar pattern to the water but were not directly associated with the water itself.

### Implications

Although the PH was placed on the U.S. EPAs National Priorities List for remediation in 2000, most measurements of PCB concentrations have focused on sediment. Little is known about the potential water fluxes and airborne PCB levels surrounding this site, and most studies have concentrated on sediment analysis. There have not been direct measurements of airborne PCBs, which is also an important source of PCBs in the communities surrounding the PH. This study, a collaboration between academic researchers and community advocates – helps PHCC know the levels of PCB in the air, previously unknown, and assess the impact of the PHSS as a potential source of airborne PCB. Knowing these air concentration levels and their impact on their community can help them be better informed while working along decision makers during PH remediation. Furthermore, these measurements are a baseline for future measurement comparisons. Airborne PCB sampling reveals the presence of PCBs in the community’s atmosphere, however, concentration levels are relatively low, with a recorded maximum of 910 pg m^–3^. By comparing measured airborne PCB concentrations with predicted levels based solely on flux from PH water, we determined that additional significant sources of PCBs must exist. Our thorough congener analysis identified non-Aroclor PCBs 47 (44 + 47 + 65), PCB 51 (45 + 51), and PCB 68 in the water, along with Aroclor 1016 and 1242 identified in the atmosphere, points to other potential sources for further investigation. Furthermore, future remediation such as sediment dredging could introduce elevated levels of atmospheric PCB contamination to the PH and increase risk to community members, which leads to the need for further analysis during these remedial actions.

## Supplementary Material



## Data Availability

Data for this work is published open access in the Pangaea repository at 10.1594/PANGAEA.983837,^53^ and all “R” codes created here to analyze, model and generate plots and maps are available in the Zenodo repository at 10.5281/zenodo.17137534 ^54^ for future reuse. There is no user registration or fee requirements to download the underlying data set. For this study we used generative artificial intelligence (AI) for the use of grammatical review along with our own experience.

## Notes

Site identification and description, air-sampling collection and extraction, depuration compounds; instrument parameters, standard reference material, limit of quantification, PCB emissions calculations, Monte Carlo simulations; and Results (PDF)
