# Supplementary material for: Airborne PCB Concentrations in Portland, Oregon: Emissions and Contributions from the Portland Harbor Superfund Site
Source: ACS EST Air. 2025 Nov 13;2(12):2922–31. doi: 10.1021/acsestair.5c00244 (PMC12706737; doi:10.1021/acsestair.5c00244)
Supplement: Supplementary file 1 [file ea5c00244_si_001.pdf]

Supplementary Information

Airborne PCB Concentrations in Portland, Oregon: Emissions and Contributions from the Portland Harbor Superfund Site.

Alexis R. Slade, [1] *Andres Martinez**[1]*, Martine E. Mathieu-Campbell*[2]*, Cassie Cohen*[3]*, Shannon Lea Watkins*[4]*, and Keri C. Hornbuckle**[1]

[1]Department of Civil and Environmental Engineering, IIHR-Hydroscience and Engineering, The University of Iowa, Iowa City, IA, 52242, USA

[2]Center for Geospatial Analytics, North Carolina State University, Raleigh, NC 27695 USA

[3]Portland Harbor Community Coalition, Portland, OR 97211, USA

[4]Department of Community and Behavior Health, College of Public Health, The University of Iowa, Iowa City, IA, 52242 US

Site Identification and Description

The Portland Harbor Community Coalition (PHCC) solicited the sites selected for this study. Through several platforms, such as the use of social media, public meetings, and contacting friends of the community, the PHCC was able to secure 31 volunteers to host passive air samplers (PAS) on their property. The Portland Harbor (PH) has historic contamination of Aroclor PCBs mainly found in the sediment and water of the PH. There has been preliminary remediation of the affected areas which was completed in 2019.

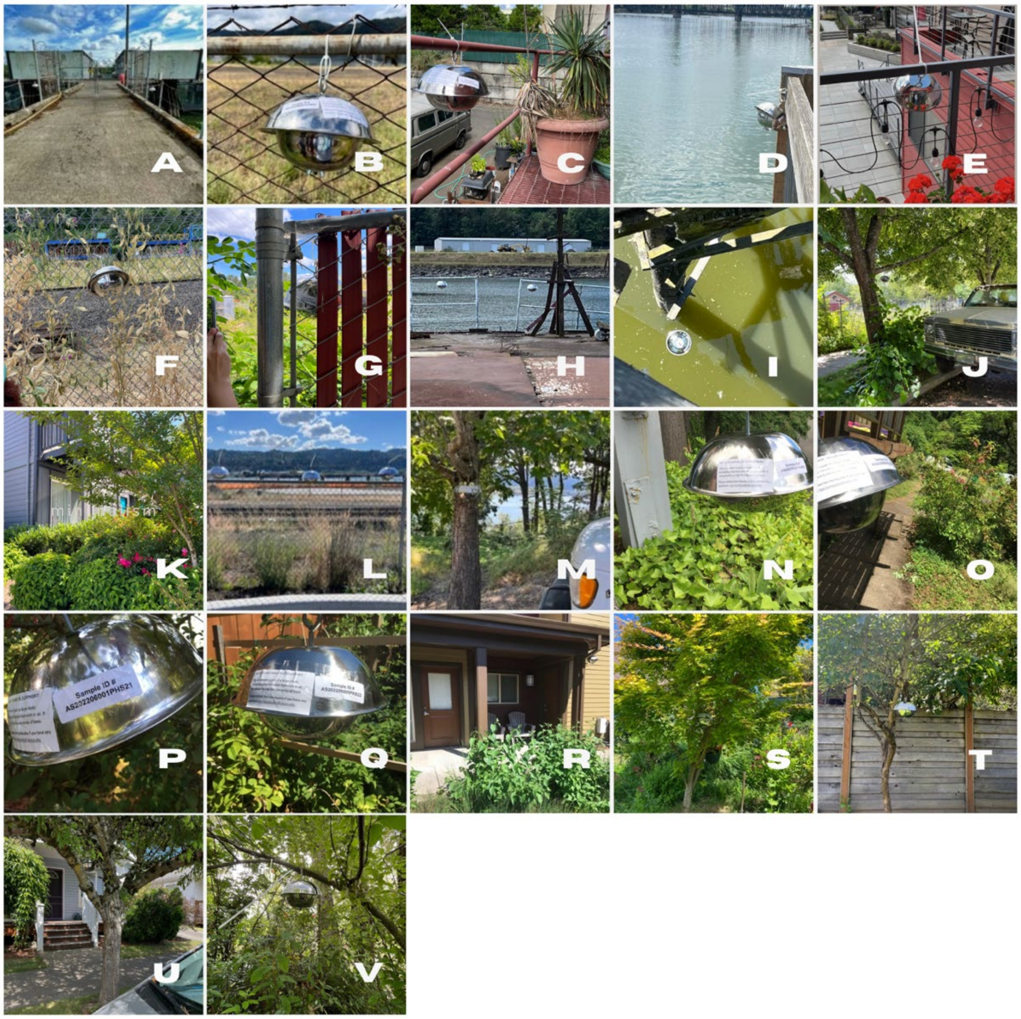

Figure S1. Sampling sites for this study were pre-identified by the PHCC based on proximity to the Portland Harbor and community volunteerism. These images depict some of the variable research environments. No images are available of site PH22: A. PH01-02; B. PH03; C. PH04; D. PH05; E. PH06; F. PH07; G. PH08; H. PH09-11; I. PH12; J. PH13; K. PH14; L. PH16-17; M. PH18; N. PH19; O. PH20; P. PH21; Q. PH23; R. PH24; S. PH28; T. PH29; U. PH30; V. PH31.

Air Sampling Collection and Extraction

Passive air samplers containing polyurethane foam were used to sample polychlorinated biphenyls (PCBs) in the atmosphere around the Portland Harbor (PH). The samplers were constructed from two stainless steel bowls, a 23 cm diameter upper bowl, and an 18 cm lower bowl. Each bowl contains holes drilled in the bottom for air circulation and to secure mounting brackets for hanging apparatus at sampling sites. The PUF disks (Tisch Environmental, USA, Part # TE-1014) are 14 cm in diameter, 1.35 cm thick, have a surface area of 365 cm^2, 207 cm^3 volume, and a density of 0.0213 g cm^{-3}. PAS are pre-cleaned with acetone and hexane solvents in the lab before deployment. PUF disks were cleaned with an ASE 350 accelerated solvent extraction system that uses acetone and hexane (1:1 v/v) for 20 minutes and then dried in a fume hood. Each PUF was individually wrapped in aluminum foil and placed in zip lock bags that were stored at -4 °C until deployment. Passive air samplers were shipped to the study location before the deployment date, and the PUF disks were transported with us on the day of deployment.

The ASE 350 maintained a pressure of 1500 psi, 100 °C, 5 min heat and static time, 100 second purge time, and a 1 cycle flush volume of 60%. To prepare the samples for extraction, 10 ng of surrogate standards containing 246 ng mL^{-1} of ^{13C}PCB 3, 15, 31, 52, 118, 153, 180, 194, 206, and 209 were added to the PUF to account for extraction efficiency, and to be used for mass corrections. The extraction process produced approximately 100 mL of sample extract. Samples were concentrated using a Turbo Vap blowdown evaporator (Biotage Turbo Vap II Automated Solvent Evaporation System) to about 0.5 mL. The concentrate was cleaned using a column packed with approximately 2 cm of quartz wool, 0.1 g of heat-treated silica, and 1 g of acidified silica. Samples were concentrated a second time to obtain the final extract, transferred to 2 mL

gas chromatography (GC) vials, and spiked with 10 ng of internal standard d-PCB 30 and PCB 204 to obtain the congener-specific masses of all 209 PCBs. The GC operated in solvent vent injection mode under the following injection conditions: initial temperature of 45 °C for 0.06 min, ramping at 600 °C min^{-1} to an inlet temperature of 325 °C at 4.4 psi. The GC oven temperature program was as follows: 45 °C for 2 min, then increased to 75 °C at 100 °C min^{-1} and held for 5 min, then to 150 °C at 15 °C min^{-1} and held for 1 min, followed by an increase to 280 °C at 2.5 °C min^{-1} with a final hold of 5 min (total run time: 70.86 min). The triple quadrupole MS electron ionization source was set to 260 °C.

Depuration Compounds

All samplers were deployed over the same period in Portland but were placed in many different situations that affected their sampling rate (R_s) and overall effective volume (V_{eff}, m^3). R_s are heavily dependent on wind speed and temperature and these rates can be different between environments. [1,2] V_{eff} is a product of the R_s and deployment time and requires consideration of non-linear uptake. We considered a scenario where the recovery of depuration compounds (DCs) was used to assess site-specific R_s and V_{eff}. [3,4] Concentrations of the DC, boundary layer calculations, equilibrium partitioning coefficient, density, volume, temperature, and time were used for R_s calculations. The variables used for R_s can be found in **Table S2**:

$$R_s = -ln\frac{(\frac{C}{C_0}) * \delta_{film} * K_{PUF} * A_{PUF}}{t}$$

C/C_0 are the DC concentrations (ng) before deployment and after, respectively. δ_{film} is the boundary layer film above the PUF (m^2), K_{PUF} is the PUF-air equilibrium partitioning coefficient (m^3 g^{-1}), A_{PUF} is the area of the PUF disk (m^2). Further details on these calculations can be found elsewhere.[2,5]

Table S1. Variable inputs for Sampling Rate calculations using eq S1.

**Meteorological Variables**	**Values**
Temperature	24 (°C)
Deployment time	42 (days)
PUF Variables	
PUF Volume	2.07E-04 (m^3)
PUF Area	3.65E-02 (m^2)
PUF Density	21300 (g m^{-3})
Constants	
Gas Constant (R)	8.31 (J/mol*K)
dU	-78380 (unitless)
Log Koa	7.60 (unitless)

The V_{eff} is then calculated as:

$$V_{eff} = (V_{PUF}K_{PUF})\left[1 - e^{-\left(\frac{R_S}{V_{PUF}K_{PUF}}\right)t}\right]$$

Where V_{PUF} is the PUF volume (m^3) and t is the deployment time (days).

The utility of the DCs for determining R_s required that a significant fraction of the spiked DC be lost to volatilization during sample deployment. Only one of our DC compounds, ^{13}C-PCB 28, lost an acceptable fraction. [1, 2] As seen in other studies with similar deployment periods (40 days), only the lower chlorinated DCs experienced suitable losses because they are more volatile. [1, 4] The DC method for determining the site-specific R_s is useful because it accounts for differences in exposure conditions and other studies have found it to provide accurate R_s for samplers deployed in different areas, [1, 6] but we found in to be unreliable.

Table S2. Variable inputs for sampling rate and effective volume calculations using depuration compounds and equations S1and S2 respectively.

**Site ID**	**Rs (m^3 day^{-1})**	**V_{eff} (m^3)**
PH01	1.65	59
PH02	7.79	170
PH03	1.39	51
PH05	6.68	158
PH06	1.07	44
PH07	1.57	57
PH09	7.83	170

PH10	3.85	114
PH11	5.15	137
PH12	1.51	55
PH16	2.87	92.7
PH17	2.62	86.4
PH24	1.48	54.2

Instrument Parameters

Table S3. PCB precursor and product masses of unlabeled and deuterated calibration standards used in multiple reaction monitoring (MRM) mode on the triple quadrupole mass spectrometer. [a]

Cl homolog	Precursor Mass	Product Mass
mono	188	153.1
di	222	152.1
tri	256	186
tetra	291.9	222
penta	325.9	255.9
hexa	359.8	289.9
hepta	393.8	323.9
octa	429.8	359.8
nona	463.7	393.8
deca	497.7	427.7
D5 tri	261	191.1
13C mono	200.1	165.1
13C di	234	164.1
13C tri	268	191.8
13C tetra	304	234
13C penta	337.9	268
13C hexa	371.9	301.9
13C hepta	405.8	335.9
13C octa	429.8	359.8
13C nona	475.8	405.8
13C deca	509.7	439.8

[a] Unlabeled standards were from AccuStandard, New Haven, CT, USA. Labeled standards were from C/D/N Isotopes, Pointe-Claire, QC, Canada.

Standard Reference Material

To evaluate the quality of our analytical methods we used standard reference material (SRM, 2585, National Institutes of Standards and Technology). SRM 2585 has NIST-specific certification criteria that reports the results of its characterizations and provides information

regarding the appropriate uses of the material. We compared our results to these certified concentrations. Our analytical methods contain coeluted congeners and are shown in **Figure S2**.

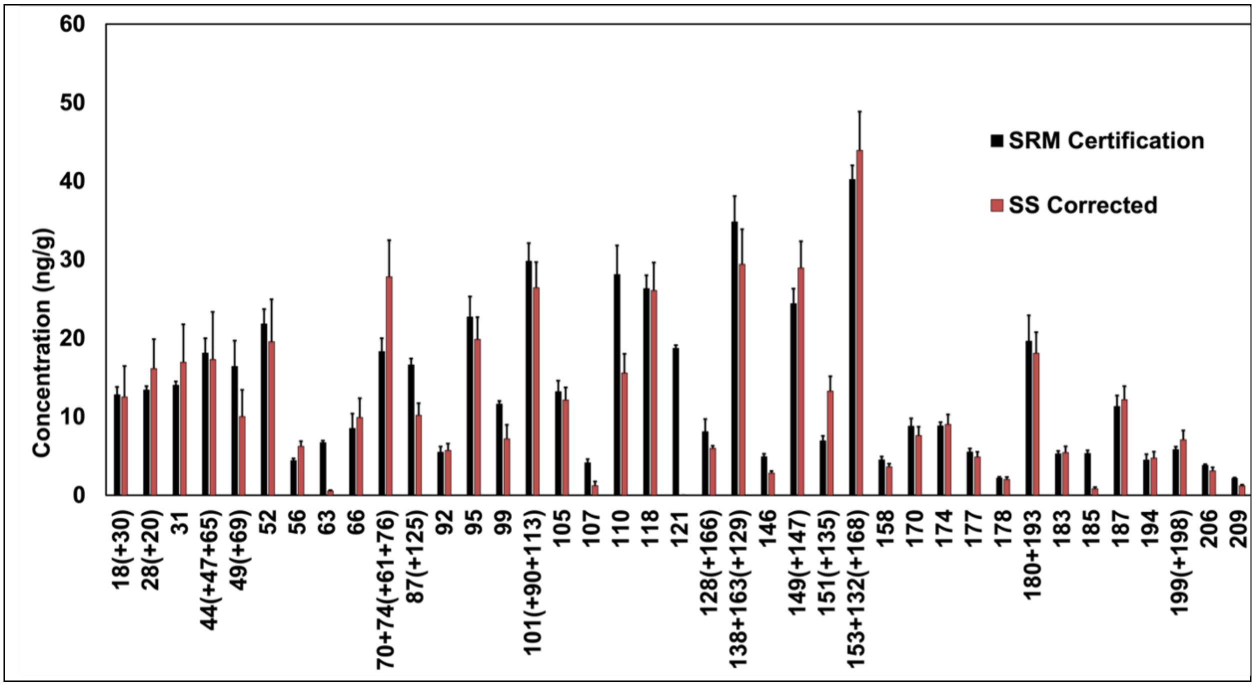

Figure S2. Analysis of National Institute of Standards and Technology Standard Reference Material (SRM) 2585, organic contaminants in house dust (n = 4).

Table S4. Evaluation of standard reference material to assess our analytical methods. SRM mean surrogate standard recoveries are reported as percentages for each congener.

PCB	Certified Values (ug/kg)	Measured Values (ug/kg)	Certified Recovery (%)
18	12.8	12.5	98%
28	13.4	16.1	120%
31	14	17	121%
44	18.1	17.3	95%
49	16.4	10	61%
52	21.8	19.5	90%
56	4.4	6.2	141%
63	6.7	0.55	8%
66	8.5	9.9	117%
70+74	18.3	27.8	152%
87	16.6	10.2	61%
92	5.5	5.7	104%
95	22.7	19.9	87%
99	11.6	7.2	62%

101	29.8	26.4	89%
105	13.2	12.1	92%
107	4.14	1.2	29%
110	28.1	15.6	55%
118	26.3	26.1	99%
121	18.7	0.0	0%
128	8.1	6.0	74%
138+163	34.8	29.4	84%
146	4.9	2.8	58%
149	24.4	28.9	119%
151	6.9	13.3	192%
153+132	40.2	43.9	109%
158	4.5	3.6	81%
170	8.8	7.6	86%
174	8.8	9.0	102%
177	5.5	4.9	89%
178	2.2	2.0	93%
180+193	19.6	18.1	92%
183	5.3	5.4	103%
185	5.3	0.8	16%
187	11.3	12.2	108%
194	4.5	4.7	106%
199	5.8	7.1	122%
206	3.8	3.1	82%
209	2.1	1.2	56%

Table S5. Mean blank and air sample surrogate standard recoveries as percentages by PCB congeners.

PCB	Mean Recovery (%)	Standard Deviation (%)
13C 3	73%	9%
13C 15	85%	11%
13C 28	91%	12%
13C 52	93%	12%
13C 118	101%	16%
13C 153	86%	13%
13C 180	85%	11%
13C 194	83%	11%
13C 206	89%	14%
13C 209	93%	19%

Limit of Quantification

A method blank was also included with each batch run to account for unwanted and unknown sources of contamination.

Table S6. Limit of quantification (LOQ) for each PCB congener or group of co-eluting congeners in units of picograms from PUF-PAS measurements. *

PCB	LOQ	PCB	LOQ	PCB	LOQ	PCB	LOQ
1	6.6	36	0.4	73	0.7	110	7.5
2	0.7	37	2.7	77	0.4	111	0.2
3	2.0	38	0.0	78	0.5	112	0.6
4	20.2	39	0.3	79	0.2	114	0.2
5	0.7	40+71	4.3	80	0.4	115	2.8
6	5.5	41	1.3	81	0.5	117	0.3
7	1.1	42	2.9	82	0.9	118	5.0
8	24.5	43	0.4	83	1.5	120	0.3
9	2.0	44+47+65	12.1	84	3.7	121	0.2
10	0.8	45	2.8	85+116	1.1	122	0.1
11	14.1	46	1.1	86+97+109+119	2.3	123	0.1
12+13	0.9	48	2.5	87+125	4.4	126	0.3
14	0.2	49+69	7.2	88	1.0	127	0.4
15	5.2	50+53	2.7	89	0.2	128+166	0.7
16	10.0	51	0.9	90+101+113	11.5	129+138+163	6.3
17	9.8	52	19.2	91	1.1	130	0.4
18+30	22.8	54	0.1	92	2.4	131	0.2
19	3.9	55	0.2	93+100	0.1	132	2.6
20+28	15.8	56	1.9	94	0.1	133	0.2
21+33	9.8	57	0.1	95	15.8	134	0.5
22	6.1	58	0.3	96	0.2	135+151	3.6
23	0.2	59+62+75	0.9	98	0.3	136	1.8
24	0.4	60	1.1	99	3.0	137	0.3
25	1.5	61+70+74+76	11.6	102	0.4	139+140	0.2
26+29	3.2	63	0.3	103	0.1	141	1.3
27	1.5	64	4.7	104	0.3	142	0.2
31	15.5	66	4.4	105	2.0	143	0.2
32	6.0	67	0.2	106	0.4	144	0.6
34	0.1	68	0.1	107	0.3	145	0.3
35	0.4	72	0.1	108+124	0.3	146	0.7
147+149	7.0	167	0.2	184	1.8	201	0.1

148	0.3	169	0.2	185	0.2	202	0.3
150	0.2	170	0.6	186	0.7	203	0.4
152	0.1	171+173	0.4	187	2.1	205	0.2
153+168	4.9	172	0.2	188	1.5	206	0.2
154	0.1	174	1.3	189	0.2	207	0.2
155	0.2	175	0.2	190	0.2	208	0.1
156+157	0.5	176	0.3	191	0.1	209	0.5
158	0.6	177	0.7	192	0.2		
159	0.2	178	0.4	194	0.3		
160	0.2	179	1.0	195	0.2		
161	0.4	180+193	1.9	196	0.3		
162	0.3	181	1.2	197	0.1		
164	0.4	182	1.7	198+199	0.6		
165	0.4	183	0.9	200	0.2		

*The LOQ was calculated as the upper limit of the 95% confidence interval of the mass in the blanks (average + 1.96 * standard deviation ÷ √12).

PCB Emissions Calculations

We calculated the gross PCB emissions from using the entire PH superfund water area of 15 km^2 for each of the 159 PCB congeners measured in the water (E_{PCBi}, ng d^{-1} m^{-2}) using the gradient-flux law, which is the product of the air-water mass transfer coefficient and the water concentration[7-10]:

$$E_{PCBi} = V_{PCBi\ a/w} \cdot C_{PCBi\ w}$$

where $V_{PCBi\ a/w}$ is the air-water mass transfer coefficient for the ith PCB (m d^{-1}) and $C_{PCBi\ w}$ is the concentration in the water column for the ith PCB (ng m^{-3}). Because the method utilized by EPA for measuring the dissolved phase of PCBs in the water does not remove the presence of dissolved organic carbon (DOC), a correction was performed, following these equations:

$$C_{PCBi\ w} = \frac{C_{PCBi\ w*}}{(1 + K_{DOC\ PCBi} \cdot [DOC])}$$

$$K_{DOC\ PCBi} = 0.06 \cdot K_{PCBi\ ow}$$

$$K_{PCBi\ ow} = K_{PCBi\ ow\ (std)} \cdot e^{(\frac{-\Delta U_{PCBi\ ow}}{R} \cdot (\frac{1}{T_w} - \frac{1}{T_{std}}))}$$

where $C_{PCBi\ w*}$ is the dissolved phase of the ith PCB measured by EPA, $K_{DOC\ PCBi}$ is the partition coefficient between dissolved organic carbon (DOC) and the freely dissolved concentration of the ith PCB[11], [DOC] is the DOC concentration (2 mg L^{-1})[12], T_{std} is the standard temperature (298.15 K), $\Delta U_{PCBi\ ow}$[13] is the internal energy for the transfer of octanol to water for the ith PCB (J mol^{-1}), $K_{PCBi\ ow\ (std)}$is the octanol-water partition coefficient at standard temperature for the ith PCB (L kg^{-1}). The mass transfer coefficient was calculated using the Whitman two-film model, in which the individual velocities across the air and water films were determined to compute the overall air-water mass transfer coefficient, as described in the following equation:

$$V_{PCBi\ a/w} = (\frac{1}{V_{PCBi\ a} \cdot K_{PCBi\ a/w}} + \frac{1}{V_{PCBi\ w}})^{-1}$$

where $V_{PCBi\ w}$ is the water mass transfer coefficient of the ith PCB (m d^{-1}), $V_{PCBi\ a}$ is the air mass transfer coefficient of the ith PCB (m d^{-1}), $K_{PCBi\ a/w}$ is the equilibrium air-water partition coefficient of the ith PCB (nondimensional Henry's Law constant) corrected by air and water temperatures. Temperature correction for the Henry's Law constant was carried out using the Van't Hoff equation[14]:

$$K_{PCBi\ a/w\ (Tstd)} = HLC_{PCBi} \cdot (\frac{1}{R \cdot T_{std}})$$

$$K_{PCBi\ a/w\ (Tw)} = K_{PCBi\ a/w\ (Tstd)} \cdot e^{(\frac{-\Delta U_{PCBi\ aw}}{R} \cdot (\frac{1}{T_w} - \frac{1}{T_{std}}))}$$

$$K_{PCBi\ a/w} = K_{PCBi\ a/w\ (Tw)} \cdot \frac{T_w}{T_a}$$

where HLC_{PCBi} is the Henry's law constant of the ith PCB (Pa m^3 mol^{-1})[15], R is the ideal gas constant (8.3144 m^3 mol^{-1} K^{-1}), Tstd is the standard temperature (298.15 K), $K_{PCBi\ a/w\ (Tstd)}$ is the nondimensional Henry's law constant of the ith PCB at standard temperature, $K_{PCBi\ a/w\ (Tw)}$

is the nondimensional Henry's law constant of the ith PCB corrected by water temperature, Tw is the water temperature (K), $\Delta U_{PCBi\ aw}$ is the internal energy for the transfer of water to air of the ith PCB (J mol^{-1}), $K_{PCBi\ a/w}$ is the water and air temperatures corrected nondimensional Henry's law constant of the ith PCB.

The mass transfer of CO_2, represented as K_{600}, varies across different water bodies. It primarily depends on two factors: the concentration gradient between surface water and air, and the physical transfer or turbulent energy at the air-water interface. For this study a K_{600} (cm h^{-1}) was obtained from Alin et. al. [16]

$$K_{600} = 4.46 + 7.11 \cdot u$$

where u is the wind speed measured at 10 meters above the surface of the water in m s^{-1}. The $V_{PCBi\ w}$ was calculated depending on the u as follow:

$$V_{PCBi\,w} = K_{600} \cdot \left(\frac{Sc_{PCBi\,w}}{Sc_{CO2}w}\right)^{-0.667} \quad u \leq 5 \left(\frac{m}{s}\right)$$
$$V_{PCBi\,w} = K_{600} \cdot \left(\frac{Sc_{PCBi\,w}}{Sc_{CO2}w}\right)^{-0.5} \quad u < 5 \left(\frac{m}{s}\right)$$

where $Sc_{PCBi\ w}$ is the Schmidt number of the ith PCB, $Sc_{CO2\ w}$ is the Schmidt number in water for CO_2. The $Sc_{CO2\ w}$ was obtained by dividing the kinematic viscosity of the water corrected by water temperature (cm^2 s^{-1}) and the CO_2 diffusivity in water corrected by water temperature (cm^2 s^{-1}), and $Sc_{PCBi\ w}$ was calculated by dividing the kinematic viscosity of the water corrected by water temperature (cm^2 s^{-1}) and the diffusivity in water of the ith PCB (cm^2 s^{-1}).

Monte Carlo Simulation

We applied a Monte Carlo approach to estimate the uncertainty in the calculated emissions. Frequency distributions for each parameter were derived from available data,

including air and water temperatures, wind speed, and atmospheric pressure, as well as from the standard deviations of reported physical-chemical property values. All parameters were assumed to follow a normal distribution. Because we used the three highest water concentration samples, we calculated the average and standard deviation for each congener. We then assumed a normal distribution, using the standard deviation as the error attributed to the sampling method and chemical analysis. The simulations were conducted 1,000 times, generating a frequency distribution of the emissions.

Results

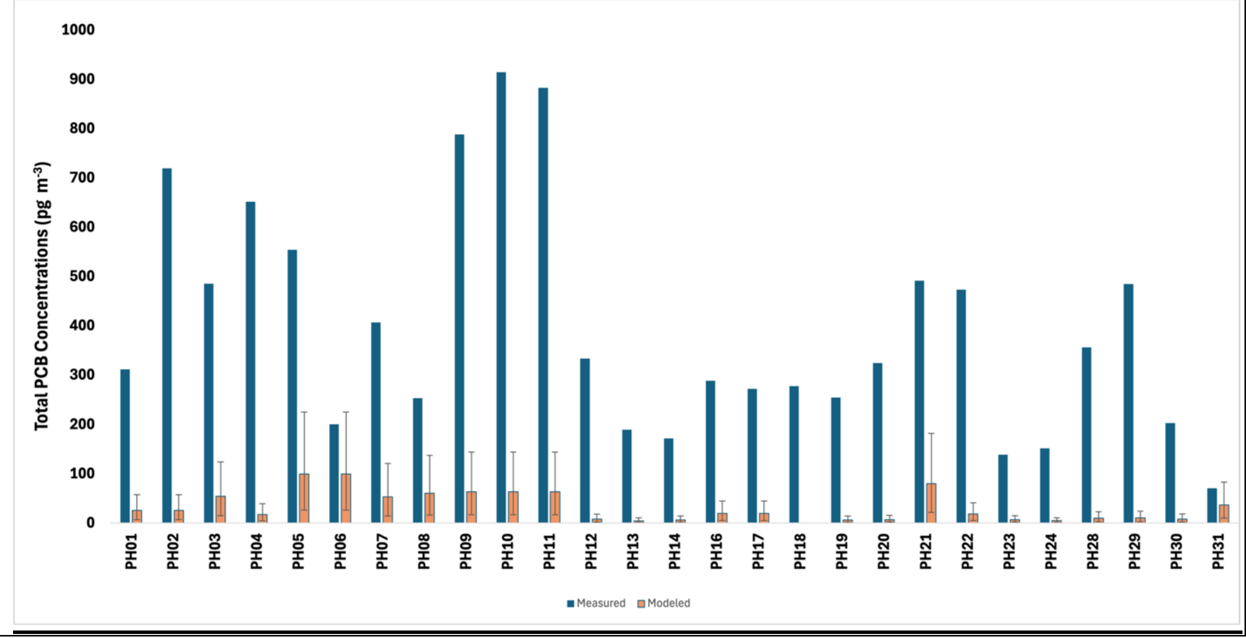

Figure S3. AERMOD predicted airborne ΣPCB concentrations vs field measurements from July to August 2022. Predictions were made using U.S. EPA water concentration data from 2018 to 2019. Predicted concentrations are shown with average plus one standard deviation and the average minus one standard deviation of the emissions.

	PH01	PH02	PH03	PH04	PH05	PH06	PH07	PH08	PH09	PH10	PH11	PH12	PH13	PH14	PH16	PH17	PH18	PH19	PH20	PH21	PH22	PH23	PH24	PH28	PH29	PH30	PH31
PH01	1.00																										
PH02	0.95	1.00																									
PH03	0.87	0.80	1.00																								
PH04	0.76	0.84	0.67	1.00																							
PH05	0.81	0.90	0.76	0.95	1.00																						
PH06	0.86	0.83	0.98	0.78	0.83	1.00																					
PH07	0.84	0.73	0.76	0.56	0.67	0.70	1.00																				
PH08	0.88	0.74	0.86	0.59	0.68	0.81	0.97	1.00																			
PH09	0.83	0.74	0.73	0.55	0.66	0.66	0.99	0.92	1.00																		
PH10	0.86	0.86	0.74	0.83	0.89	0.76	0.91	0.86	0.90	1.00																	
PH11	0.84	0.77	0.73	0.59	0.69	0.67	0.98	0.91	1.00	0.92	1.00																
PH12	0.83	0.70	0.72	0.52	0.61	0.67	0.98	0.97	0.96	0.87	0.94	1.00															
PH13	0.95	0.94	0.89	0.81	0.86	0.92	0.76	0.83	0.73	0.83	0.75	0.75	1.00														
PH14	0.91	0.81	0.93	0.69	0.78	0.91	0.91	0.97	0.87	0.88	0.87	0.90	0.91	1.00													
PH16	0.87	0.76	0.77	0.59	0.69	0.72	1.00	0.97	0.99	0.92	0.98	0.98	0.79	0.92	1.00												
PH17	0.86	0.78	0.77	0.61	0.71	0.72	0.99	0.94	0.99	0.93	0.99	0.96	0.79	0.91	0.99	1.00											
PH18	0.84	0.70	0.78	0.53	0.63	0.72	0.99	0.99	0.96	0.87	0.95	0.99	0.76	0.93	0.99	0.97	1.00										
PH19	0.90	0.81	0.84	0.67	0.78	0.81	0.98	0.97	0.96	0.94	0.96	0.96	0.85	0.96	0.98	0.98	0.97	1.00									
PH20	0.80	0.63	0.81	0.46	0.57	0.74	0.96	0.98	0.92	0.80	0.90	0.97	0.73	0.93	0.95	0.93	0.99	0.94	1.00								
PH21	0.78	0.63	0.68	0.45	0.55	0.61	0.98	0.95	0.96	0.84	0.94	0.99	0.68	0.86	0.97	0.95	0.99	0.94	0.96	1.00							
PH22	0.89	0.81	0.78	0.70	0.78	0.76	0.98	0.96	0.96	0.96	0.96	0.96	0.84	0.93	0.98	0.98	0.96	0.99	0.92	0.94	1.00						
PH23	0.84	0.81	0.97	0.76	0.82	0.99	0.70	0.81	0.65	0.75	0.66	0.66	0.92	0.91	0.71	0.71	0.71	0.80	0.73	0.59	0.76	1.00					
PH24	0.95	0.90	0.95	0.80	0.86	0.96	0.83	0.90	0.80	0.87	0.81	0.81	0.97	0.97	0.85	0.84	0.84	0.91	0.83	0.75	0.88	0.96	1.00				
PH28	0.80	0.66	0.73	0.48	0.59	0.66	0.99	0.97	0.96	0.85	0.95	0.99	0.71	0.90	0.98	0.96	0.99	0.96	0.98	0.99	0.95	0.65	0.79	1.00			
PH29	0.81	0.66	0.71	0.48	0.58	0.65	0.99	0.97	0.96	0.85	0.94	1.00	0.71	0.89	0.98	0.96	0.99	0.95	0.97	1.00	0.95	0.64	0.79	1.00	1.00		
PH30	0.85	0.71	0.82	0.56	0.67	0.77	0.97	0.99	0.93	0.87	0.93	0.98	0.78	0.96	0.97	0.95	0.99	0.97	0.99	0.96	0.95	0.76	0.87	0.98	0.98	1.00	
PH31	0.89	0.81	0.95	0.72	0.81	0.94	0.87	0.94	0.82	0.86	0.82	0.85	0.91	0.99	0.88	0.87	0.89	0.93	0.90	0.81	0.90	0.94	0.97	0.85	0.84	0.93	1.00

Figure S4. Cosine theta similarity results for air samples in Portland, Oregon around the Portland Harbor Superfund Site.

	PH01	PH02	PH03	PH04	PH05	PH06	PH07	PH08	PH09	PH10	PH11	PH12	PH13	PH14	PH16	PH17	PH18	PH19	PH20	PH21	PH22	PH23	PH24	PH28	PH29	PH30	PH31
1221 A1	0.24	0.14	0.18	0.09	0.11	0.18	0.32	0.36	0.27	0.23	0.26	0.39	0.22	0.34	0.32	0.31	0.37	0.29	0.37	0.35	0.29	0.18	0.26	0.37	0.38	0.38	0.32
1232 A1.5	0.52	0.39	0.42	0.27	0.34	0.38	0.69	0.68	0.65	0.56	0.64	0.72	0.45	0.62	0.68	0.67	0.71	0.64	0.68	0.70	0.64	0.39	0.51	0.72	0.72	0.70	0.58
1232 G1.5	0.52	0.39	0.42	0.26	0.33	0.38	0.68	0.67	0.64	0.55	0.63	0.71	0.44	0.61	0.67	0.67	0.70	0.63	0.68	0.69	0.63	0.38	0.50	0.71	0.72	0.69	0.57
1016 A2	0.65	0.57	0.58	0.36	0.49	0.48	0.90	0.79	0.94	0.78	0.94	0.85	0.52	0.71	0.89	0.91	0.86	0.85	0.82	0.88	0.84	0.47	0.61	0.88	0.87	0.82	0.66
1016 S2	0.65	0.57	0.58	0.36	0.49	0.48	0.90	0.79	0.94	0.78	0.94	0.85	0.52	0.71	0.89	0.91	0.86	0.85	0.82	0.88	0.84	0.47	0.61	0.88	0.87	0.82	0.66
1242 A3	0.72	0.65	0.61	0.47	0.58	0.54	0.91	0.81	0.94	0.83	0.95	0.86	0.60	0.75	0.91	0.92	0.86	0.88	0.81	0.87	0.88	0.55	0.67	0.87	0.87	0.83	0.70
1242 G3	0.69	0.62	0.60	0.44	0.55	0.52	0.90	0.79	0.93	0.81	0.94	0.83	0.57	0.72	0.89	0.91	0.84	0.86	0.79	0.85	0.85	0.52	0.64	0.86	0.84	0.81	0.68
1242 S3B	0.72	0.64	0.61	0.46	0.58	0.54	0.92	0.82	0.94	0.83	0.95	0.87	0.60	0.76	0.91	0.92	0.87	0.88	0.82	0.88	0.88	0.54	0.67	0.89	0.88	0.84	0.71
1248 A3.5	0.63	0.70	0.52	0.66	0.74	0.55	0.62	0.54	0.65	0.74	0.69	0.54	0.60	0.59	0.64	0.67	0.54	0.68	0.47	0.50	0.68	0.56	0.61	0.53	0.52	0.54	0.59
1248 G3.5	0.59	0.66	0.48	0.60	0.67	0.50	0.57	0.49	0.60	0.68	0.64	0.49	0.55	0.53	0.59	0.62	0.50	0.62	0.42	0.45	0.62	0.52	0.56	0.48	0.47	0.49	0.54
1254 A4	0.48	0.53	0.34	0.46	0.46	0.40	0.24	0.27	0.23	0.34	0.25	0.22	0.58	0.34	0.26	0.27	0.21	0.32	0.14	0.14	0.35	0.43	0.45	0.16	0.18	0.20	0.33
1254 G4	0.60	0.71	0.44	0.69	0.67	0.54	0.32	0.35	0.32	0.52	0.34	0.30	0.71	0.45	0.36	0.37	0.29	0.42	0.20	0.21	0.47	0.55	0.59	0.23	0.25	0.28	0.45
1260 A5	0.50	0.56	0.29	0.29	0.28	0.30	0.12	0.17	0.15	0.20	0.17	0.15	0.49	0.22	0.16	0.17	0.13	0.18	0.08	0.11	0.18	0.26	0.37	0.09	0.11	0.12	0.21
1260 S5	0.49	0.55	0.28	0.29	0.28	0.29	0.12	0.17	0.15	0.20	0.17	0.15	0.48	0.22	0.16	0.17	0.13	0.18	0.08	0.11	0.17	0.26	0.36	0.09	0.11	0.12	0.21
1260 G5	0.48	0.54	0.27	0.27	0.26	0.28	0.11	0.16	0.14	0.18	0.16	0.14	0.47	0.20	0.15	0.15	0.12	0.17	0.07	0.10	0.16	0.24	0.35	0.08	0.10	0.11	0.19
1262 A6	0.37	0.41	0.20	0.17	0.18	0.20	0.09	0.12	0.11	0.12	0.12	0.11	0.37	0.16	0.12	0.12	0.09	0.13	0.06	0.09	0.11	0.17	0.27	0.07	0.08	0.08	0.14
1262 G6	0.37	0.41	0.20	0.17	0.18	0.20	0.08	0.12	0.10	0.12	0.12	0.10	0.37	0.16	0.11	0.12	0.09	0.12	0.06	0.08	0.11	0.17	0.27	0.06	0.07	0.08	0.14

Figure S5. Cosine theta similarity results for air samples and Aroclors around the Portland Harbor Superfund Site.

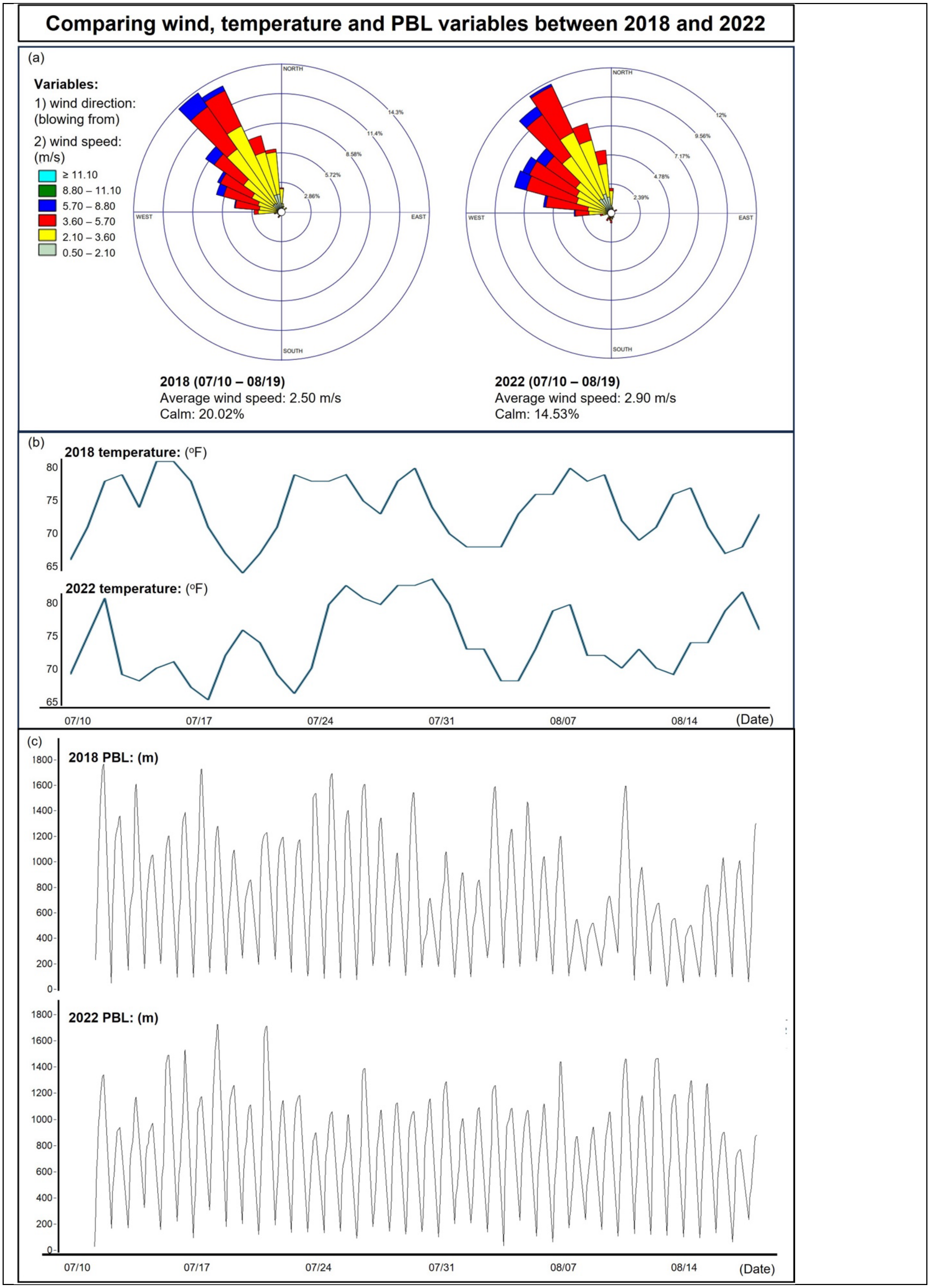

Comparing wind, temperature and PBL variables between 2018 and 2022
(a)
Variables:
1) wind direction: (blowing from)
2) wind speed: (m/s)
≥ 11.10
8.80 – 11.10
5.70 – 8.80
3.60 – 5.70
2.10 – 3.60
0.50 – 2.10
NORTH
SOUTH
WEST
EAST
14.3%
11.4%
8.58%
5.72%
2.86%
12%
9.56%
7.17%
4.78%
2.39%
2018 (07/10 – 08/19)
Average wind speed: 2.50 m/s
Calm: 20.02%
2022 (07/10 – 08/19)
Average wind speed: 2.90 m/s
Calm: 14.53%
(b)
2018 temperature: (°F)
2022 temperature: (°F)
80
75
70
65
07/10
07/17
07/24
07/31
08/07
08/14
(Date)
(c)
2018 PBL: (m)
2022 PBL: (m)
1800
1600
1400
1200
1000
800
600
400
200
0

Figure S6. Comparison between temperature, wind parameters, and PBL during the same period in 2018 and 2022: a) wind rose plots presenting wind speed and wind direction; b) time series of temperature; c) daily average PBL height.

Table S1. Measured versus modeled PCB Concentrations (pg m^{-3}).

Site ID	Measured	Modeled	Ratio
PH01	312	25	8%
PH02	720	25	3%
PH03	485	55	11%
PH04	652	17	3%
PH05	555	99	18%
PH06	200	99	50%
PH07	407	53	13%
PH08	253	61	24%
PH09	788	64	8%
PH10	914	64	7%
PH11	883	64	7%
PH12	334	8	2%
PH13	189	5	3%
PH14	172	6	4%
PH16	289	20	7%
PH17	273	20	7%
PH18	278	20	7%
PH19	255	6	2%
PH20	325	7	2%
PH21	491	80	16%
PH22	473	18	4%
PH23	138	7	5%
PH24	152	5	3%
PH28	356	10	3%
PH29	485	11	2%
PH30	203	8	4%
PH31	70	37	53%

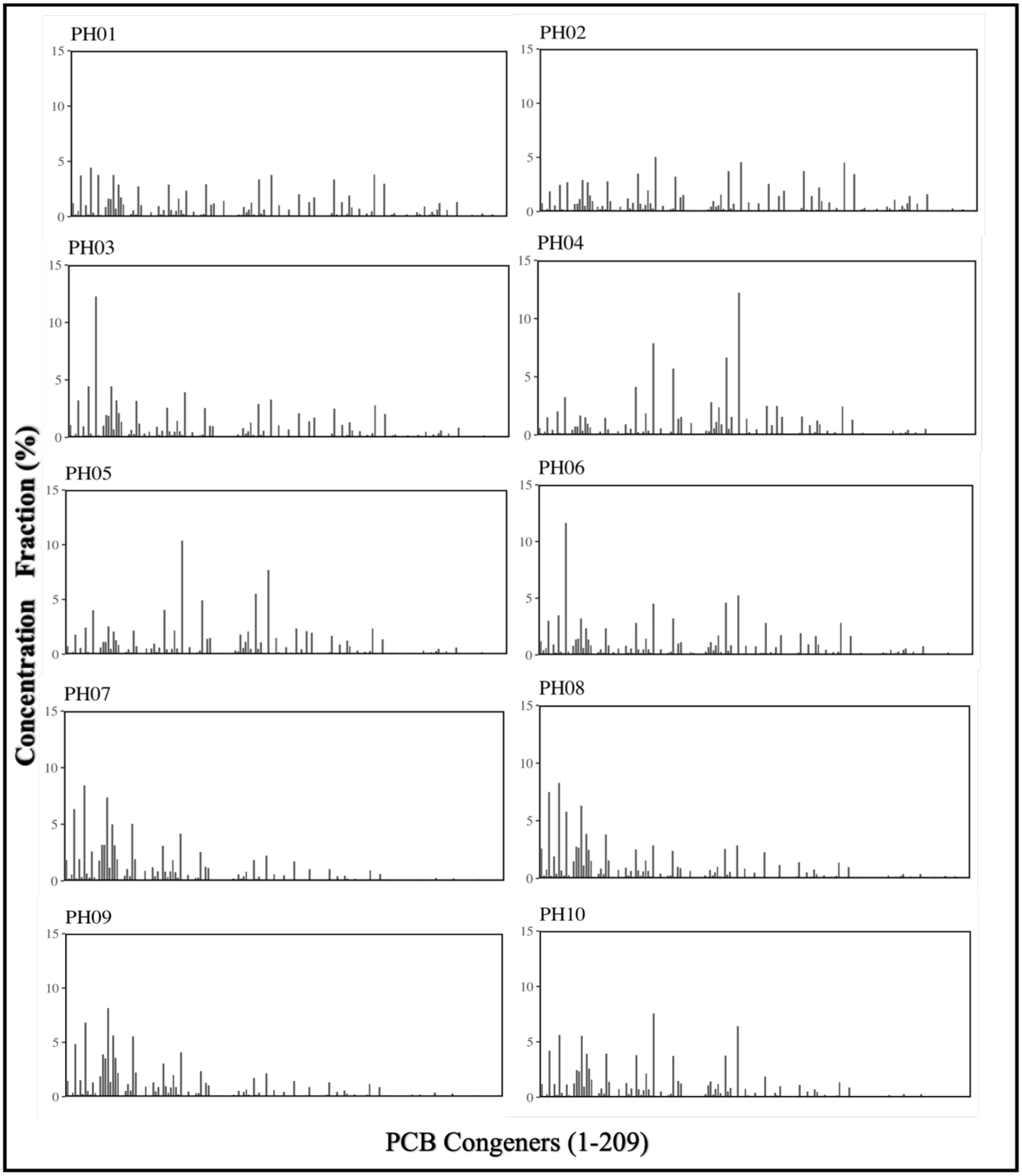
PH01
PH02
PH03
PH04
PH05
PH06
PH07
PH08
PH09
PH10
Concentration Fraction (%)
PCB Congeners (1-209)

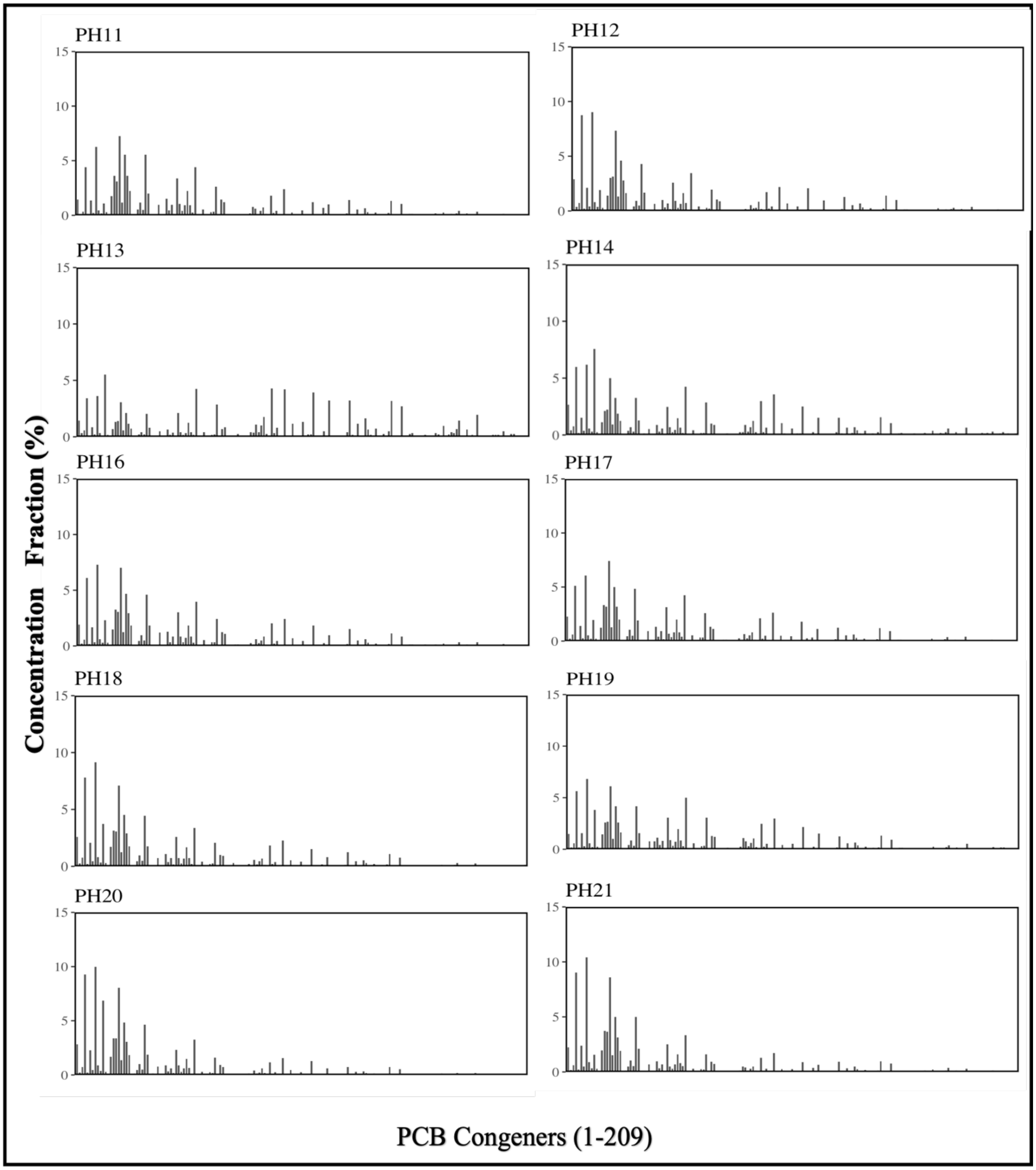
PH11
PH12
PH13
PH14
PH16
PH17
PH18
PH19
PH20
PH21
0
5
10
15
Concentration Fraction (%)
PCB Congeners (1-209)

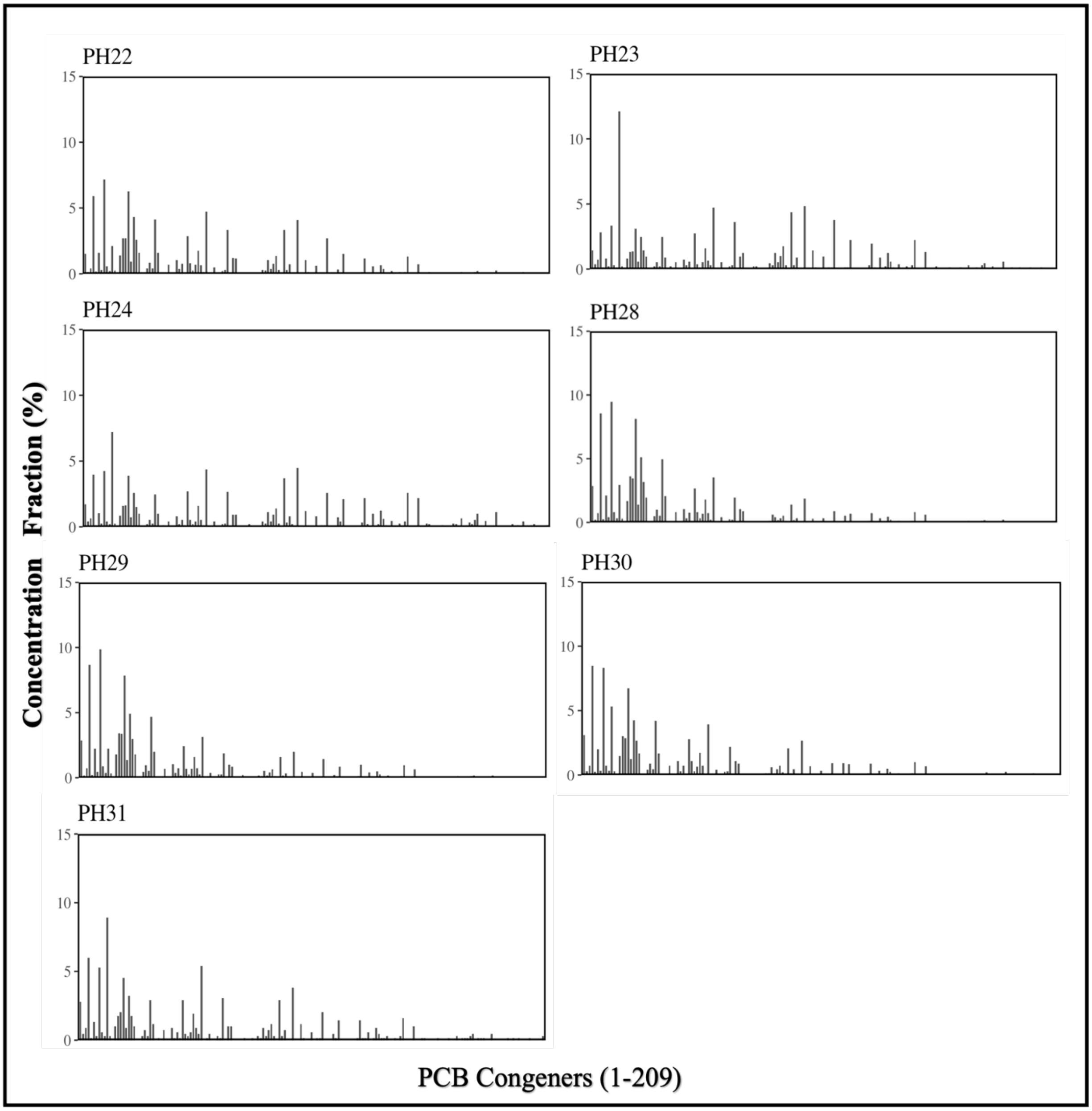

Figure S7. PCB congener profiles for each individual site sampled at the Portland Harbor. Profiles are in concentration fraction of ΣPCB (n=27).

REFERENCES

(1) Moeckel, C.; Harner, T.; Nizzetto, L.; Strandberg, B.; Lindroth, A.; Jones, K. C. Use of Depuration Compounds in Passive Air Samplers: Results from Active Sampling-Supported Field Deployment, Potential Uses, and Recommendations. *Environ. Sci. Technol* **2009**, *43* (9), 3227-3232. DOI: 10.1021/es802897x.
(2) Wania, F.; Shunthirasingham, C. Passive air sampling for semi-volatile organic chemicals. *Environmental science--processes & impacts* **2020**, *22* (1), 1925-1922. DOI: 10.1039/d0em00194e.
(3) Bartkow, M. E.; Jones, K. C.; Kennedy, K. E.; Holling, N.; Hawker, D. W.; Müller, J. F. Evaluation of performance reference compounds in polyethylene-based passive air samplers. *Environ Pollut* **2006**, *144* (2), 365-370. DOI: 10.1016/j.envpol.2005.12.043.
(4) Persoon, C.; Hornbuckle, K. C. Calculation of passive sampling rates from both native PCBs and depuration compounds in indoor and outdoor environments. *Chemosphere* **2009**, *74* (7), 917-923. DOI: 10.1016/j.chemosphere.2008.10.011.
(5) Herkert, N. J.; Spak, S. N.; Smith, A.; Schuster, J. K.; Harner, T.; Martinez, A.; Hornbuckle, K. C. Calibration and evaluation of PUF-PAS sampling rates across the Global Atmospheric Passive Sampling (GAPS) network. *Environ Sci Process Impacts* **2018**, *20* (1), 210-219. DOI: 10.1039/c7em00360a.
(6) Pozo, K.; Harner, T.; Lee, S. C.; Wania, F.; Muir, D. C. G.; Jones, K. C. Seasonally Resolved Concentrations of Persistent Organic Pollutants in the Global Atmosphere from the First Year of the GAPS Study. *Environmental Science & Technology* **2009**, *43* (3), 796-803. DOI: 10.1021/es802106a.
(7) Martinez, A.; Wang, K.; Hornbuckle, K. C. Fate of PCB Congeners in an Industrial Harbor of Lake Michigan. *Environ. Sci. Technol* **2010**, *44* (8), 2803-2808. DOI: 10.1021/es902911a.
(8) Schwarzenbach, R. P. Environmental organic chemistry / René P. Schwarzenbach, Philip M. Gschwend, Dieter M. Imboden. **2003**.
(9) Totten, L. A.; Gigliotti, C. L.; Offenberg, J. H.; Baker, J. E.; Eisenreich, S. J. Reevaluation of Air−Water Exchange Fluxes of PCBs in Green Bay and Southern Lake Michigan. *Environ. Sci. Technol* **2003**, *37* (9), 1739-1743. DOI: 10.1021/es026093x.
(10) Zhang, H. X.; Eisenreich, S. J.; Franz, T. R.; Baker, J. E.; Offenberg, J. H. Evidence for increased gaseous PCB fluxes to Lake Michigan from Chicago. *Environmental Science & Technology* **1999**, *33* (13), 2129-2137. DOI: 10.1021/es981073+.
(11) Burkhard, L. P. Estimating dissolved organic carbon partition coefficients for nonionic organic chemicals. *Environmental Science & Technology* **2000**, *34* (22), 4663-4668. DOI: 10.1021/es001269l.
(12) Spencer, R. G. M.; Butler, K. D.; Aiken, G. R. Dissolved organic carbon and chromophoric dissolved organic matter properties of rivers in the USA. *Journal of Geophysical Research: Biogeosciences* **2012**, *117* (G3). DOI: https://doi.org/10.1029/2011JG001928.
(13) Li, N. Q.; Wania, F.; Lei, Y. D.; Daly, G. L. A comprehensive and critical compilation, evaluation, and selection of physical-chemical property data for selected polychlorinated biphenyls. *Journal of Physical and Chemical Reference Data* **2003**, *32* (4), 1545-1590. DOI: 10.1063/1.1562632.
(14) Goss, K. U. Prediction of the temperature dependency of Henry's law constant using poly-parameter linear free energy relationships. *Chemosphere* **2006**, *64* (8), 1369-1374. DOI: 10.1016/j.chemosphere.2005.12.049.

(15) Dunnivant, F. M.; Elzerman, A. W.; Jurs, P. C.; Hasan, M. N. Quantitative structure-property relationships for aqueous solubilities and Henry's law constants of polychlorinated biphenyls. *Environ. Sci. Technol* **1992**, *26* (8), 1567-1573. DOI: 10.1021/es00032a012.
(16) Alin, S. R.; Rasera, M.; Salimon, C. I.; Richey, J. E.; Holtgrieve, G. W.; Krusche, A. V.; Snidvongs, A. Physical controls on carbon dioxide transfer velocity and flux in low-gradient river systems and implications for regional carbon budgets. *Journal of Geophysical Research-Biogeosciences* **2011**, *116*. DOI: 10.1029/2010jg001398.
